# Self-Assembly Properties of Xylene-Derived Constitutional Isomers of Fmoc-Phenylalanine

**DOI:** 10.1021/acs.langmuir.5c02581

**Published:** 2025-09-18

**Authors:** Pamela Agredo, Ritty Mohan, Sydney T. Carter, Bradley L. Nilsson

**Affiliations:** † Department of Chemistry, 6927 University of Rochester, Rochester, New York 14627-0216, United States; ‡ Materials Science Program, University of Rochester, Rochester, New York 14627-0166, United States

## Abstract

Fluorenylmethoxycarbonyl-phenylalanine (Fmoc-Phe) derivatives are a privileged molecular class that readily undergoes supramolecular self-assembly into hydrogel networks. Herein, we characterize the self-assembly properties of xylene-derived constitutional isomers of Fmoc-Phe, demonstrating the impact of molecular configuration on the emergent structure and properties of supramolecular assemblies of these derivatives. The self-assembly properties of Fmoc-Phe and a cationic derivative of Fmoc-Phe that has been modified at the C terminus with diaminopropane (DAP), Fmoc-Phe-DAP, were compared to those of several corresponding xylene derivatives in which Fmoc-functionalized amine and carboxylic acid or DAP-functionalized carboxylic acid are organized around a central benzene ring, with the appended functionality oriented in *ortho*, *meta*, or *para* spatial arrangements. Under conditions where Fmoc-Phe and Fmoc-Phe-DAP derivatives undergo self-assembly into fibrillar supramolecular hydrogel networks, it was found that corresponding xylene derivatives assemble into distinctive nanoribbon/nanotape morphologies that fail to form supramolecular networks that elicit emergent hydrogel formation. The assemblies formed are dependent on the spatial arrangement of the xylene core structure. These studies provide insight into the significant effects of molecular arrangement on the supramolecular self-assembly properties of constitutional isomers of phenylalanine derivatives.

## Graphical abstract

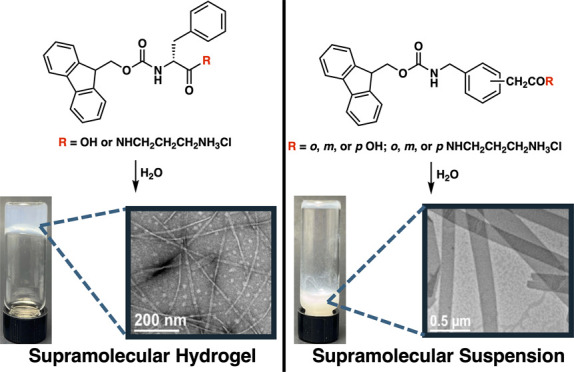

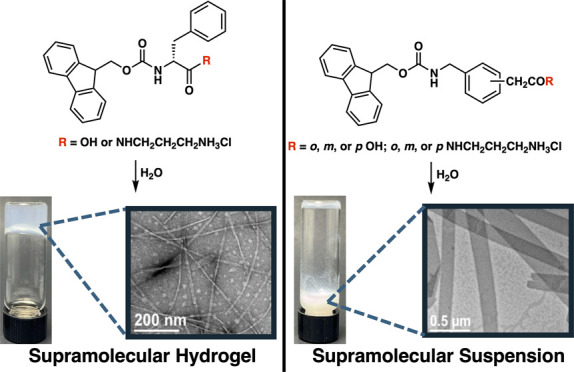

## Introduction

The supramolecular self-assembly of low-molecular-weight (LMW) amino acid derivatives has inspired the development of novel, next-generation biomaterials, including hydrogels for drug delivery, regenerative medicine, and tissue engineering.^1–17^ Fluorenylmethoxycarbonyl-phenylalanine (Fmoc-Phe; Figure 1) and its derivatives are especially prone to self-assemble into hydrogel networks in water.^18–31^ The self-assembly of Fmoc-Phe derivatives is driven by a delicate balance between attractive and repulsive forces, including hydrophobic and π–π interactions, hydrogen bonding, and both attractive and repulsive charge effects.^32–44^ Notably, even minor modifications in the Fmoc-Phe derivative structure can significantly influence the assembly properties of these derivatives. For example, changing the position of a single halogen substituent on the benzyl side chain group of Fmoc-Phe derivatives dramatically impacts the assembly morphology, the formation of hydrogel networks, and the emergent viscoelastic properties of the resulting networks.^45,46^ Despite extensive empirical research, predicting the self-assembly characteristics of Fmoc-Phe derivatives from molecular structure alone is a significant unmet challenge. Further, the correlation between molecular structure and the emergent hydrogel properties of supramolecular Fmoc-Phe assemblies is poorly understood.

**Figure 1:**
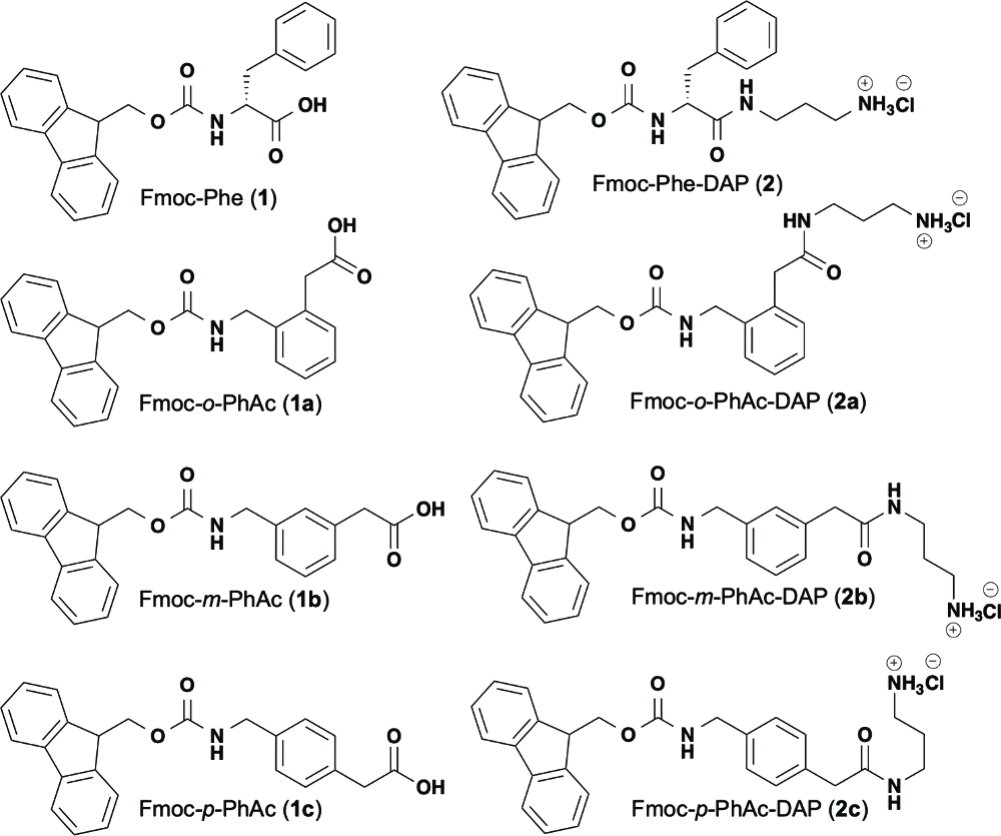
Chemical structures of Fmoc-Phe (**1**) and Fmoc-Phe-DAP (**2**) isomers: Fmoc-*ortho*-aminomethyl-phenylacetic acid (Fmoc-*o*-PhAc) (**1a**), Fmoc-*meta*-aminomethyl-phenylacetic acid (Fmoc-*m*-PhAc) (**1b**), Fmoc-*para*-aminomethyl-phenylacetic acid (Fmoc-*p*-PhAc) (**1c**), Fmoc-*o*-PhAc-DAP (**2a**), Fmoc-*m*-PhAc-DAP (**2b**), and Fmoc-*p*-PhAc-DAP (**2c**).

Efforts to address this challenge include extensive structure–function analyses that characterize the relationship between the self-assembling properties of novel Fmoc-Phe derivatives and the molecular structure of these derivatives.^21,45,47–49^ These studies include the analysis of constitutional isomers of Fmoc-Phe. For example, we have previously compared the self-assembly of Fmoc-Phe derivatives to their corresponding peptoid isomers in which the benzyl side chain has been shifted from α carbon to amino nitrogen.^50^ While the parent amino acid derivatives self-assembled into fibril hydrogel networks, the peptoid analogs instead favored assembly into crystalline sheets due to the lack of N–H hydrogen bond donors.

Here, we report an additional comparative analysis of the self-assembly properties of xylene-derived constitutional isomers of Fmoc-Phe (Figure 1). Specifically, we investigate Fmoc-aminomethyl-phenylacetic acid (Fmoc-PhAc) derivatives that have identical molecular formulas and functional group content as Fmoc-Phe but differ in the three-dimensional configuration of the functional groups. Fmoc-Phe derivatives present the benzyl side chain group, the Fmoc-modified nitrogen, and the carboxylic acid organized from central stereogenic α carbon. In contrast, Fmoc-PhAc derivatives feature the benzene group as the central organizing functional group from which Fmoc-nitrogen and carboxylic acid are presented in various relative orientations, *ortho*, *meta*, or *para*. The Fmoc-PhAc derivatives can participate in the same intermolecular interactions as the corresponding Fmoc-Phe derivatives, although these interactions are constrained by the differing geometric configurations that are accessible based on the constraints of the Fmoc-PhAc structures. These studies provide significant insight into the relationship between molecular structure and self-assembly properties of constitutional isomers of Fmoc-Phe.

## Experimental Section

### Materials

Reagents and organic solvents were purchased commercially and used without further purification. The synthetic protocol and characterization of compounds **2a**–**2c** are reported below. NMR spectra and mass spectral data are shown in Figures S1–S9. Water used for gelation and solution preparation was purified using a nanopure filtration system (Barnstead NANOpure, 0.2 μm filter, 18.2 MΩ cm).

### NMR Spectroscopy

NMR spectroscopy to characterize synthetic compounds was performed using a Brüker Avance 500 MHz spectrometer. ^1^H, and ^13^C chemical shifts are reported as δ with reference to TMS at 0 ppm for ^1^H, and residual solvent for ^13^C. See Figures S1–S9 of the Supporting Information for NMR spectra and mass spectra. Tabulated data for all synthetic compounds is also listed in the Supporting Information.

### Mass Spectrometry

High-resolution mass spectra for compounds **2a**, **2b**, and **2c** were obtained on a Thermo Fisher Q Exactive Plus Hybrid Quadrupole-Orbitrap mass spectrometer in positive mode. See the tabulated mass spectra data and mass spectra (Figures S3, S6, and S9) for these compounds in the Supporting Information.

### Self-Assembly by pH Adjustment for Anionic Compounds **1**, **1a**, **1b**, and **1c**

Compounds were dissolved in basic water (15 mM NaOH) at concentrations of 10 mM to allow dissolution. A fresh solution of glucono-δ-lactone (GdL) was prepared at a concentration of 100 mg/mL (561 mM) immediately prior to gelation. Assemblies were prepared by adding 1 mol equiv of the GdL solution into the compound solution (10 mM final concentration of GdL). The solution was mixed by brief agitation by vortex after the addition of GdL and left undisturbed for approximately 24 h to allow self-assembly. pH was measured using an 89231-592 pH probe (VWR Symphony), 6.5 mm diameter. Digital images of all assemblies at 24 h after GdL addition are provided.

### Self-assembly by increasing ionic strength for cationic compounds **2**, **2a**, **2b**, and **2c**

All compounds were assembled with a final gelator concentration of 10 mM, final NaCl concentration of 0, 10, 25, or 114 mM, and total volume of 1 mL. Compounds (0.02 mmol) were dissolved in 0.8 mL of nanopure water at 70 °C in a glass vial, followed by sonication until complete dissolution. Then, 0.2 mL of an aqueous NaCl solution was added to the vial, which was immediately and briefly agitated by a vortex mixer. The samples were allowed to stand for 1 h, then the vial was inverted to determine if the sample was a self-supporting hydrogel. pH was measured using an 89231-592 pH probe (VWR Symphony) with a 6.5 mm diameter. Digital images of all assemblies at 24 h after salt addition are provided.

### Transmission Electron Microscopy (TEM)

TEM images were obtained using a Hitachi 7650 transmission electron microscope with an accelerating voltage of 80 kV. Aliquots of assembled materials (5 μL) were applied directly onto 200 mesh carbon-coated copper grids and allowed to stand for 1 min. Excess sample was carefully removed by capillary action using filter paper, and the grids were then stained with 2% (w/v) uranyl acetate (5 μL) for 2 min. Excess stain was removed by capillary action, and the grids were allowed to air-dry for 10 min. Dimensions of the network structures were determined using ImageJ software and are reported as the average of at least 100 independent measurements, with error reported as the standard deviation about the mean.

### Quantitative NMR Measurements

For quantitative NMR to determine the ratio of assembled and unassembled derivatives in water, all compounds (0.02 mmol) were dissolved in 0.8 mL of D_2_O in a glass vial by heating and sonication, as described above. Then, this solution was placed into an NMR tube, and the self-assembly was triggered with the addition of glucono-δ-lactone (GdL) for carboxylic acid derivatives (**1** and **1a**–**1c**) or NaCl (**2** and **2a**–**2c**) as described in the self-assembly protocols above. Also, reference solutions of all compounds in DMSO-*d*_6_ and D_2_O (10 mM) were prepared as standards for unassembled states. NMR tubes were fitted with an internal capillary containing 24 mM DMF in DMSO-*d*_6_ as an external standard to assist quantification. The percentage of assembly was measured by comparative integration of signal peaks of the DMSO-*d*_6_ sample for each compound. Each signal was integrated relative to the external DMF standard.

### Fourier Transform Infrared (FTIR) Spectroscopy

ATR–FTIR spectra were measured using a PerkinElmer Spectrum 3 spectrometer equipped with a diamond ATR accessory. Spectra were collected from 4000–400 cm^–1^ at 2 cm^–1^ resolution, averaging 124 scans. Data were recorded in absorption mode and baseline corrected before analysis. Samples were prepared in two forms: (i) neat powders of the carboxylic acid derivatives **1** and **1a**–**1c**, the corresponding DAP derivatives **2** and **2a**–**2c**, and (ii) lyophilized samples of corresponding assemblies and hydrogels of each derivative. Spectra were measured for both the powders and the lyophilized gel samples to compare the molecular organization before and after gelation.

### Experimental Log *P* Partition Coefficient Determinations

Experimental log *P* partition coefficients for each compound were determined using the reported stir-flask method.^51^ 5 mL of *n*-octanol and 5 mL of water were added to a 50 mL round-bottomed flask. The mixtures were sealed and stirred continuously for 48 h at room temperature to achieve mutual saturation of the phases. Compounds were added to the flask to final concentrations between 0.5 and 5 mM, and then stirred continuously for 24 h at the same temperature. The mixtures were then centrifugated in 50 mL Falcon tubes for 3 min to allow complete separation of the layers. Finally, the UV spectrum of each layer was measured using a Shimadzu UV-2401PC UV–visible spectrophotometer, and the log *P* values were calculated using eq 1. Each experiment was performed in triplicate, with error reported as the standard deviation about the mean.

1 $$\log P=\log\left(\frac{C_{n\mathrm{‐octanol}}}{C_{\mathrm{water}}}\right)$$

## Results and Discussion

Two sets of Fmoc-PhAc derivatives that correlate to the previously characterized anionic Fmoc-Phe (**1**) and cationic diaminopropane-modified Fmoc-Phe-DAP (**2**) are reported in this study (Figure 1). These include Fmoc-Phe isomers, Fmoc-*o*-PhAc (**1a**), Fmoc-*m*-PhAc (**1b**), Fmoc-*p*-PhAc (**1c**); and Fmoc-Phe-DAP isomers, Fmoc-*o*-PhAc-DAP (**2a**), Fmoc-*m*-PhAc-DAP (**2b**), Fmoc-*p*-PhAc-DAP (**2c**). Compounds **1a**–**1c** were purchased commercially, and compounds **2a**–**2c** were synthesized and characterized as described in the Materials and Methods and Figures S1–S9 of the Supporting Information. The Fmoc-Phe and Fmoc-Phe-DAP isomers differ in the method used to promote self-assembly. The anionic Fmoc-Phe derivatives (compounds **1** and **1a**–**1c**) are dissolved in basic water and undergo self-assembly upon gradual pH adjustment by the addition of glucono-δ-lactone (GdL), which slowly hydrolyses in water to give gluconic acid, decreasing the pH of the system and protonating the carboxylate groups, thus reducing repulsive charge effects and promoting self-assembly.^52,53^ The cationic Fmoc-Phe-DAP isomers (compounds **2** and **2a**–**2c**) undergo self-assembly in water upon the addition of NaCl, which increases the solution ionic strength, thus reducing charge repulsion between ammonium cations and facilitating self-assembly.^54,55^ To further understand the impact of molecular configuration on self-assembly properties of Fmoc-Phe derivatives, we characterized the fibril morphology, the degree of self-assembly, and the emergent properties of each system. Each compound underwent self-assembly to various degrees, but none of the xylene derivatives formed fibril assemblies that resulted in stable emergent hydrogel networks. In addition, the morphology of the assemblies formed from each configurational isomer were unique, demonstrating the integral relationship between chemical structure and emergent self-assembly properties for these supramolecular systems. These results are described in detail below.

The self-assembly properties of Fmoc-Phe (**1**) were first compared to those of Fmoc-*o*-PhAc (**1a**), Fmoc-*m*-PhAc (**1b**), and Fmoc-*p*-PhAc (**1c**). To initiate self-assembly, each derivative was dissolved at 10 mM concentrations in basic water (15 mM NaOH). Subsequently, a glucono-δ-lactone (GdL) solution was added to gradually decrease the pH,^56,57^ resulting in partial protonation of the free carboxylates of Fmoc-Phe and xylene derivatives **1a**–**1c**, initiating self-assembly. Identical assembly conditions, including GdL concentration, were employed for all derivatives. The final solution pH for these derivatives after GdL hydrolysis ranged from 6.5 to 8.6 (see Table S1 of the Supporting Information for the pH of all solutions before and 24 h after GdL addition and hydrolysis). Before GdL addition, Fmoc-Phe and compounds **1a**–**1c** were homogeneous, optically transparent solutions. After GdL hydrolysis (24 h), the Fmoc-Phe solution formed a self-supporting, nearly optically transparent hydrogel composed of a supramolecular fibril network, as previously reported (Figure 2A).^52^ In contrast, xylene isomers **1a**–**1c** formed opaque colloidal suspensions that were significantly more viscous than the initial basic solutions, but that did not form self-supporting hydrogel networks (Figure 2B–D insets). TEM imaging of these suspensions revealed that Fmoc-*o*-PhAc (**1a**) assembled into narrow 10 nm diameter nanofibrils (Figure 2B). Fmoc-*m*-PhAc (**1b**) formed assemblies composed of clusters of pseudocrystalline aggregates ∼190 nm in width (Figure 2C). Fmoc-*p*-PhAc (**1c**) assembled into polymorphic nanoribbon sheets with average diameters of ∼60 nm. While xylene isomers **1a**–**1c** have the same aromatic, hydrogen bond donor/acceptor, and carboxyl group functionality as Fmoc-Phe, the varied geometric presentation of these functional groups, including the *ortho*, *meta*, and *para* substitution patterns around the central benzene scaffold, result in distinct assemblies for each xylene derivative (these assemblies are tabulated for comparison in Table S2 of the Supporting Information). The xylene derivative assemblies are morphologically unable to form stable hydrogel networks under these assembly conditions.

**Figure 2:**
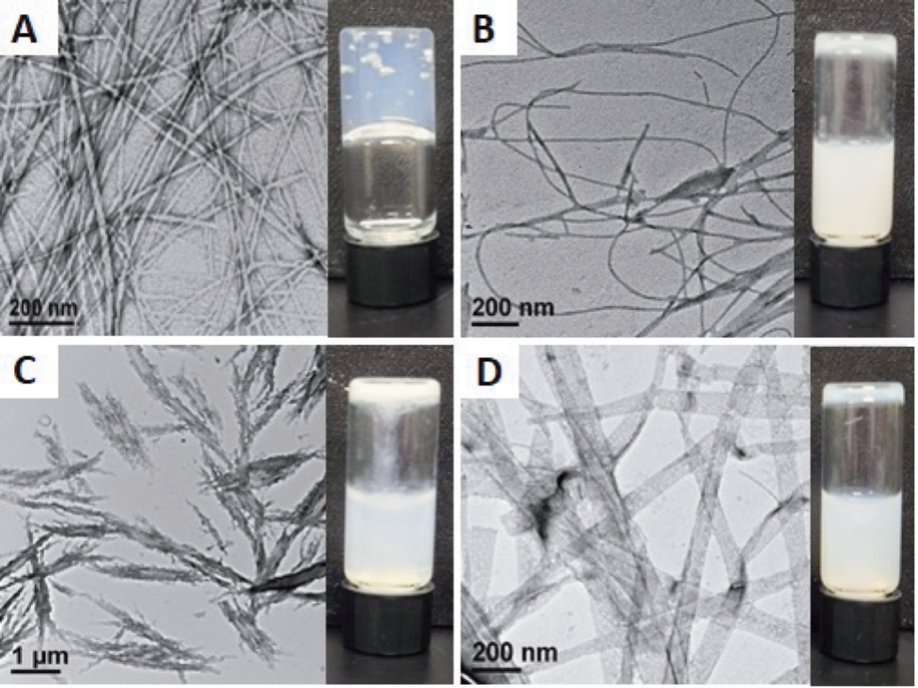
TEM and digital images of 10 mM solutions of (A) Fmoc-Phe (**1**), (B) Fmoc-*o*-PhAc (**1a**), (C) Fmoc-*m*-PhAc (**1b**), and (D) Fmoc-*p*-PhAc (**1c**) 24 h after addition of 10 mM GdL to promote self-assembly by gradual acidification.

Solution-state ^1^H NMR spectroscopy was employed to quantify the extent of monomer incorporation into the self-assembled structures for Fmoc-Phe (**1**) and xylene derivatives **1a**–**1c**. In solution-state NMR, signals of self-assembled materials disappear due to anisotropic line broadening. Thus, comparative integration of selected peaks against an external standard allows quantification of the degree of assembly.^54^ Fmoc-Phe (**1**) and xylene isomers **1a**–**1c** were each dissolved at 10 mM concentrations in DMSO-*d*_6_ (unassembled), D_2_O with 15 mM NaOH (partially assembled), and D_2_O with equimolar GdL (assembled), 10 mM in NMR tubes. ^1^H NMR spectra were obtained for each sample with an external standard of dimethylformamide (DMF) to facilitate quantitative comparative integration (see Figure S10 of the Supporting Information for spectra and the Materials and Methods for detailed protocols). Interestingly, all compounds showed some degree of assembly under basic conditions in order **1a** (41% monomer) > **1c** (53% monomer) > Fmoc-Phe (**1**) (63% monomer) > **1b** (83% monomer) (Table 1). Upon incubation with GdL for 24 h, the trend follows the order **1a** (12% monomer) > Fmoc-Phe (**1**) (25% monomer) > **1c** (40% monomer) > **1b** (45% monomer). These trends indicate that the substitution pattern of the xylene derivatives strongly influences the self-assembly propensity, with *ortho* > *para* > *meta* both before and after pH adjustment with GdL. In the absence of high-resolution structural data for the packing structure of the correlated assemblies, the reasons for these trends are not readily evident. Interestingly, the parent Fmoc-Phe (**1**), the only derivative to form an emergent hydrogel, falls into the middle of these trends. This suggests that the self-assembly propensity alone does not determine the formation of hydrogel networks. Instead, the morphology of the assembled structures must more closely correlate with emergent hydrogelation.

**Table 1:** Quantification of the Degree of Assembly of Fmoc-Phe (**1**) and Xylene-Derived Isomers (**1a**–**1c**) by Comparative Integration of ^1^H NMR Signals against an External Standard^a^

observed unassembled monomer concentration				
solvent	Fmoc-Phe (**1**) (%)	Fmoc-*o*-PhAc (**1a**) (%)	Fmoc-*m*-PhAc (**1b**) (%)	Fmoc-*p*-PhAc (**1c**) (%)
DMSO-*d*_6_	100	100	100	100
D_2_O/NaOH	63	41	83	53
D_2_O/NaOH and GdL (10 mM)	25	12	45	40

a It is assumed that all compounds are 100% unassembled in DMSO-*d*_6_, and data suggest that compounds in D_2_O are partially assembled, with the highest degree of assembly observed after acidification with GdL.

We have previously shown that modification of the Fmoc-Phe carboxylate with cationic diaminopropane (Fmoc-Phe-DAP, **2**) enables self-assembly and hydrogelation of aqueous solutions upon increases in ionic strength by the addition of NaCl or other salts.^3,54,55,58^ As with the Fmoc-Phe derivatives described above, we also compared the self-assembly of Fmoc-Phe-DAP (**2**) with the corresponding xylene derivatives, Fmoc-*o*-PhAc-DAP (**2a**), Fmoc-*m*-PhAc-DAP (**2b**), and Fmoc-*p*-PhAc-DAP (**2c**) (Figure 1). These compounds were dissolved in water by heating suspensions to 70 °C and allowing them to cool to room temperature. Subsequently, a solution of concentrated NaCl was added to final concentrations of 10 mM Fmoc-Phe-DAP or xylene derivative and 114 mM NaCl. The addition of brine triggered the self-assembly of these molecules. The pH of these unbuffered solutions ranged from 5.4 to 6.4 in water, and upon NaCl addition increased slightly to ∼6.9–7.1, bringing the solutions close to neutrality (Table S3 of the Supporting Information). Notably, after NaCl addition, the pH values of the xylene isomers (**2a**–**2c**) and the parent Fmoc-Phe-DAP (**2**) were all close to neutral (∼6.9–7.1), with only minor differences among the derivatives. This indicates that differences in self-assembly propensity cannot be attributed to variations in protonation state.

Self-assembly of the cationic Fmoc-Phe-DAP (**2**) and xylene derivatives (**2a**–**2c**) was initially characterized by visual observation of the solutions and TEM imaging of the resulting assemblies after the addition of NaCl. As previously reported, Fmoc-Phe-DAP (**2**) rapidly (seconds) forms self-supporting hydrogels composed of twisted nanoribbons (Figure 3A; see Table S4 of the Supporting Information for tabulated dimensions of assemblies). Over extended time periods (days–weeks), these nanoribbons mature into nanotubes.^54,59^ In comparison, Fmoc-*o*-PhAc-DAP (**2a**) formed an opaque, viscous suspension composed of nanotubes (∼220 nm wide) and nanosheets (Figure 3B and Table S4 of the Supporting Information). Fmoc-*m*-PhAc-DAP (**2b**) self-assembled into thin nanofibers (∼12 nm in diameter) (Figure 3C and Table S4 of the Supporting Information). Solutions of Fmoc-*p*-PhAc-DAP (**2c**) were opaque, viscous suspensions composed of formed two-dimensional nanoribbon/nanosheets (∼200 nm wide) (Figure 3D and Table S4 of the Supporting Information). None of the xylene-derived isomers formed emergent self-supporting hydrogels, even though increases in solution viscosity were observed. In addition to these studies at 10 mM compound, we also tested a range of concentrations to estimate the critical gelation concentration for Fmoc-Phe-DAP (**2**) and to determine if compounds **2a**–**2c** formed hydrogels at higher concentrations (Figure S11 of the Supporting Information). The critical gelation concentration for Fmoc-Phe-DAP (**2**) is between 1 and 2 mM in 114 mM NaCl (Figure S11A of the Supporting Information). Under these same solvent conditions, derivatives **2a**–**2c** failed to form hydrogels even at concentrations as high as 20 mM (Figure S11B–D of the Supporting Information), indicating that the assemblies formed from these xylene derivatives do not possess the necessary structural properties to form emergent hydrogel networks.

As with Fmoc-Phe (**1**) and the corresponding xylene derivatives (**1a**–**1c**), ^1^H NMR was also used to quantify the free monomer concentration of Fmoc-Phe-DAP (**2**) and xylenes **2a**–**2c** before and after NaCl addition. NMR experiments were conducted at 10 mM concentrations of compounds **2**, **2a**, **2b**, or **2c** in DMSO-*d*_6_ (unassembled), D_2_O, and D_2_O with 114 mM NaCl (Figures S12–S15 of the Supporting Information). As shown in Table 2, compounds **2**, **2a**, **2b**, and **2c** are practically entirely assembled after the addition of 114 mM NaCl, with 2% of **2**, 0.4% of **2a**, 0.3% of **2b**, and 0.1% of **2c** detectable. It is notable that the xylene derivatives **2a**–**2c** exhibit more complete assembly than Fmoc-Phe-DAP, even though these assemblies do not form hydrogel networks. Interestingly, the solutions in D_2_O with no added NaCl also showed a reduction in the amount of unassembled monomer detected, with Fmoc-Phe-DAP (**2**) showing 63% free monomer, and compounds **2a**, **2b**, and **2c** displaying 21, 28, and 9% free monomer, respectively. The xylene DAP derivatives have a significantly higher propensity to self-assemble under these conditions as well. Because these derivatives were appreciably unassembled even without explicitly added NaCl, we also conducted NMR experiments with only one molar equivalent of NaCl (10 mM) added and observed that under these conditions, the amount of observable unassembled compound was intermediate between no salt and 114 mM NaCl. Specifically, at 10 mM NaCl the observed unassembled monomer was 36% for **2**, 9% for **2a**, 10% for **2b**, and 2% for **2c**. The xylene isomers were thus shown to have a high self-assembly propensity than the corresponding Fmoc-Phe-DAP parent molecule under all conditions.

**Table 2:** Quantification of Degree of Assembly of Fmoc-Phe-DAP (**2**) and Xylene-Derived Isomers (**2a**–**2c**) by Comparative Integration of ^1^H NMR Signals against an External Standard^a^

observed unassembled monomer concentration				
solvent	Fmoc-Phe-DAP (**2**) (%)	Fmoc-*o*-PhAc-DAP (**2a**) (%)	Fmoc-*m*-PhAc-DAP (**2b**) (%)	Fmoc-*p*-PhAc-DAP (**2c**) (%)
DMSO-*d*_6_	100	100	100	100
D_2_O	63	21	28	9
D_2_O + NaCl (114 mM)	2	0.4	0.3	0.1
D_2_O + NaCl (10 mM)	36	9	10	2

a It is assumed that all compounds are 100% unassembled in DMSO-*d*_6_, and data indicates that compounds in D_2_O are partially assembled, with the highest degree of assembly observed after addition of 114 mM NaCl. Quantification was also performed in samples with lower concentrations of NaCl (10 mM).

We conducted TEM analyses to characterize the assembly morphology of each DAP derivative (10 mM) under three conditions: no added salt (Figure S16 of the Supporting Information), 10 mM NaCl (Figure S17 of the Supporting Information), and 25 mM NaCl (Figure S18 of the Supporting Information). Fmoc-Phe-DAP (**2**) formed self-supporting hydrogels under all the experimental conditions. The morphology of its assemblies remained essentially unchanged, consistently forming nanotapes or nanoribbons (Table S4 of the Supporting Information). The xylene-DAP derivatives (**2a**–**2c**) did not form self-supporting hydrogels at high salt concentration (114 mM NaCl; Figure 3). Interestingly, Fmoc-*m*-PhAc-DAP (**2b**) formed weak hydrogels in the absence of salt (Figure S16C of the Supporting Information) and at 25 mM NaCl (Figure S18C of the Supporting Information). However, these gels were unstable and reverted to solutions upon mild agitation. All three xylene-DAP isomers generally exhibited increased viscosity and opacity upon salt addition. When examining the morphology of Fmoc-*o*-PhAc-DAP (**2a**), we observed only minor morphological differences under varying conditions. It formed narrow nanotubes with diameters of ∼180 nm in water without salt (Figure S16B of the Supporting Information), ∼130 nm at 10 mM NaCl (Figure S17B of the Supporting Information), and ∼190 nm at 25 mM NaCl (Figure S18B of the Supporting Information), slightly smaller than under high salt conditions (114 mM NaCl, ∼200 nm). Similarly, nanosheets of Fmoc-*p*-PhAc-DAP (**2c**) exhibited only minor differences in the sheet dimensions while maintaining similar overall morphology at varying salt concentrations (Figures S16B, S17B, and S18B of the Supporting Information). Collectively, these studies demonstrate that Fmoc-Phe-DAP (**2**), Fmoc-*o*-PhAc-DAP (**2a**), Fmoc-*m*-PhAc-DAP (**2b**), and Fmoc-*p*-PhAc-DAP (**2c**) form distinctive assembled structures as a function of differences in chemical structure and that only the parent Fmoc-Phe-DAP (**2**) assemblies have the characteristic of emergent hydrogel viscoelastic character. The xylene derivatives, **2a**–**2c**, have assembly equilibria that are more favorable for assembly than for the parent phenylalanine.

**Figure 3:**
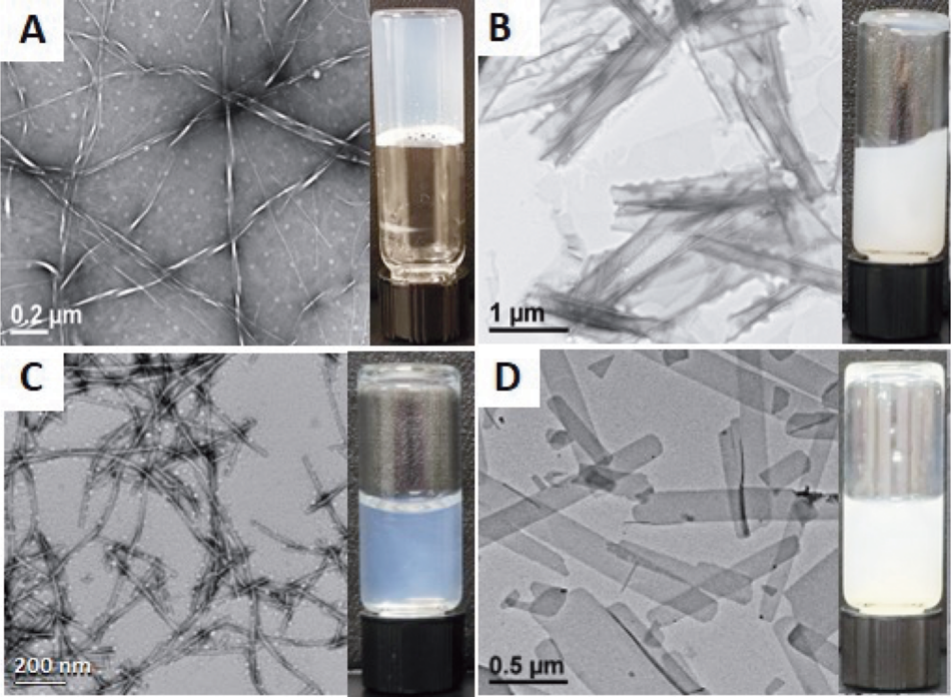
TEM images and digital images of (A) Fmoc-Phe-DAP (**2**), (B) Fmoc-*o*-PhAc-DAP (**2a**), (C) Fmoc-*m*-PhAc-DAP (**2b**), and (D) Fmoc-*p*-PhAc-DAP (**2c**) at 10 mM in aqueous brine (114 mM NaCl).

To evaluate the molecular assembly of these compounds, FTIR spectra of Fmoc-Phe (**1**), its phenylacetic acid derivatives (**1a**–**1c**; Figures S19–S22 of the Supporting Information), and the corresponding DAP derivatives (**2** and **2a**–**2c**; Figures S23–S26 of the Supporting Information) were recorded for both neat powders and xerogels derived from lyophilized samples from each hydrogel and assembly. In all cases, characteristic amide I (∼1650–1690 cm^–1^) and amide II (∼1530–1545 cm^–1^) signals were observed. Upon gelation, these bands shifted (∼1650 → 1630 cm^–1^ and ∼1540 → 1530 cm^–1^) and broadened, reflecting changes in hydrogen bonding and backbone interactions. Additional differences in the aromatic region (∼1600 and 1500 cm^–1^) and fingerprint region (∼1200–1000 cm^–1^) indicated reduced crystallinity and altered supramolecular packing. These results confirm molecular reorganization upon self-assembly in each of these samples. While these data demonstrate molecular rearrangements consistent with self-assembly, they do not support extrapolation of a specific packing architecture for these assemblies.

Finally, we also experimentally characterized the hydrophobicity of each Fmoc-Phe and xylene derivative in this study by measuring their partition coefficients (*P*) expressed as log *P* values (Figure 4). These experiments were used to determine whether significant differences in hydrophobicity between these compounds, despite their molecular similarity, may account for observed differences in self-assembly behavior, particularly self-assembly propensity. Log *P* is the logarithmic of the partition coefficient (*P*), which is the concentration ratio of a molecule between two immiscible liquids, usually water and *n*-octanol, and it is a physicochemical parameter commonly used to measure hydrophobicity.^60,61^ We performed these measurements using the stir-flask method (see the Supporting Information for experimental details).^51^ The values are reported in Table S5 of the Supporting Information. Negative log *P* values correspond to net hydrophilic character, positive values correspond to hydrophobic character, and values close to 0 indicate that the molecule is amphipathic.^51^ The Fmoc-Phe derivatives have the following log *P* values: Fmoc-Phe (1.24), Fmoc-*o*-PhAc (4.13), Fmoc-*m*-PhAc (4.42), Fmoc-*p*-PhAc (4.74). The Fmoc-Phe-DAP derivatives have the following log *P* values: Fmoc-Phe-DAP (**2**) (−0.103), Fmoc-*o*-PhAc-DAP (**2a**) (−0.411), Fmoc-*m*-PhAc-DAP (**2b**) (−0.557), Fmoc-*p*-PhAc-DAP (**2c**) (−0.759). Interestingly, the self-assembly propensities, as determined by quantitative NMR, inversely correlate with the hydrophobicity trends for the Fmoc-Phe-DAP series. The Fmoc-Phe-DAP derivatives increase in self-assembly propensity in the order **2** < **2a** < **2b** < **2c**. The order of hydrophobicity for these derivatives increases in the order **2c** < **2b** < **2a** < **2**. For the Fmoc-Phe series, the derivatives increase in self-assembly propensity in the order **1b** < **1c** < **1** < **1a**, while the hydrophobicity increases in the order **1** < **1a** < **1b** < **1c**. In contrast to the Fmoc-Phe-DAP series, the self-assembly propensity for the Fmoc-Phe derivatives does not strongly correlate with the observed hydrophobicity. This allows us to conclude that the differences in assembly propensity and assembly morphologies between these various derivatives are more likely due to inherent effects in the three-dimensional presentation of the functional groups than to effects arising from the relative hydrophobicity of these compounds.

**Figure 4:**
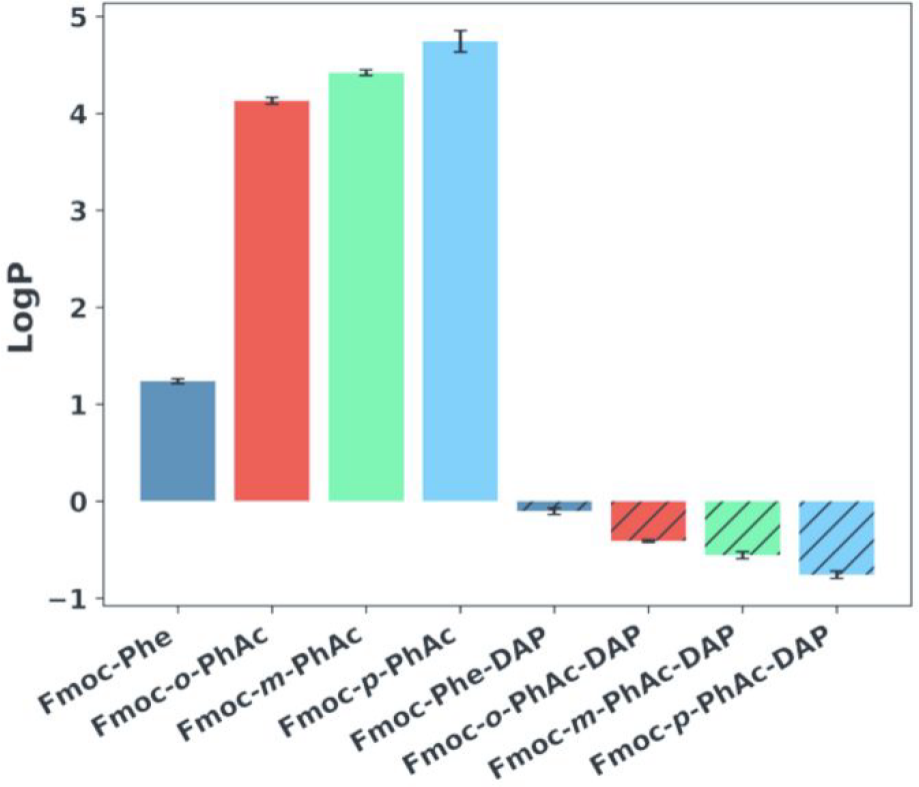
Log *P* values for Fmoc-Phe (**1**), Fmoc-Phe-DAP (**2**), and corresponding xylene derivatives.

## Conclusion

In conclusion, this study provides significant insight into the relationships between molecular structure, self-assembly character, and the emergent properties of the resulting supramolecular assemblies. Despite decades of intense research focused on elucidating the molecular principles that define supramolecular materials, these relationships remain poorly understood. In this study, we have examined the self-assembly properties of constitutional isomers of the privileged Fmoc-Phe scaffold. Two sets of constitutional isomers of anionic Fmoc-Phe (**1**) and cationic Fmoc-Phe-DAP (**2**) were studied in which a xylene scaffold presents the same functional groups present on the parent Phe-derivatives, benzene, Fmoc-amine, and carboxyl, in differing three-dimensional configurations based on the *ortho*, *meta*, and *para* substitution patterns of the central xylene core. The xylene derivatives lack the stereogenic centers found in Fmoc-Phe and Fmoc-Phe-DAP. In addition, the geometry of these xylene derivatives places the benzene functionality in a central molecular position as opposed to the peripheral orientation observed in the parent Fmoc-Phe derivatives.

These studies provide several key insights. First, the xylene derivatives were all found to self-assemble in water with more favorable critical concentrations for self-assembly than the parent Fmoc-Phe derivatives. Second, the morphologies of these xylene assemblies are distinct for each substitution pattern and distinct from the assemblies formed from the parent Fmoc-Phe molecules. Third, none of the xylene derivative assemblies form stable hydrogel networks as do both Fmoc-Phe and Fmoc-Phe-DAP. Based on similarities in chemical structure and hydrophobicity, it is unsurprising that the xylene isomers of Fmoc-Phe and Fmoc-Phe-DAP exhibit robust self-assembly properties. However, the variety of assembly structures formed, which include nanofibrils, nanoribbons/nanosheets, and nanotubes, is remarkable. It was also unexpected that none of these assemblies formed emergent hydrogel networks. We have previously observed that fibrillar assemblies tend to be best suited to the formation of emergent hydrogels, although this is not strictly required. Thus, the variety of observed assembly morphologies of the xylene isomers likely precludes the formation of these networks. In addition, differences in the packing structures are likely to alter the surface structure of the assemblies, which may result in hydrophobic functionality at the surface of the assemblies in the xylene isomers that would otherwise be buried within fibrils of Fmoc-Phe and Fmoc-Phe-DAP, making these more likely to engage in network formation in water. The lack of high-resolution packing structures for these assemblies makes it impossible to draw these conclusions currently. Thus, in addition to the novel insights these studies provide into the integral relationship between molecular structure and corresponding supramolecular properties, these studies also illustrate ongoing challenges to understanding these effects precisely. Additional studies that focus on elucidating self-assembled molecular structures as a function of molecular composition promise to bridge ongoing gaps in understanding.^62,63^

## Supplementary Material



## Notes

Supporting data, including NMR spectra of all synthetic compounds (PDF)
