# Supplementary material for: Self-Assembly Properties of Xylene-Derived Constitutional Isomers of Fmoc-Phenylalanine
Source: Langmuir. 2025 Sep 18;41(38):25962–9. doi: 10.1021/acs.langmuir.5c02581 (PMC12490004; doi:10.1021/acs.langmuir.5c02581)
Supplement: Supplementary file 1 [file la5c02581_si_001.pdf]

Supporting Information

Self-Assembly Properties of Xylene-Derived Constitutional Isomers of Fmoc-Phenylalanine

*Pamela Agredo,[a] Ritty Mohan,[a] Sydney T. Carter,[a] and Bradley L. Nilsson[a,b]**

[a] Department of Chemistry, University of Rochester, Rochester, NY, 14627-0216, USA

[b] Materials Science Program, University of Rochester, Rochester, NY 14627-0166, USA

E-mail: bradley.nilsson@rochester.edu

Tel. +1 585 276-3053

Supporting Information

Table of Contents

Synthesis of Fmoc-Phe-DAP isomers S4

Figure S1. ^{1}H NMR spectrum of Fmoc-o-PhAc-DAP (2a).. S7

Figure S2. ^{13}C NMR spectrum of Fmoc-o-PhAc-DAP (2a)... S8

Figure S3. High-resolution mass spectrum of Fmoc-o-PhAc-DAP (2a)..................................... S9

Figure S4. ^{1}H NMR spectrum of Fmoc-m-PhAc-DAP (2b). S10

Figure S5. ^{13}C NMR spectrum of Fmoc-m-PhAc-DAP (2b). S11

Figure S6. High-resolution mass spectrum of Fmoc-m-PhAc-DAP (2b). S12

Figure S7. ^{1}H NMR spectrum of Fmoc-p-PhAc-DAP (2c).. S13

Figure S8. ^{13}C NMR spectrum of Fmoc-p-PhAc-DAP (2c).. S14

Figure S9. High-resolution mass spectrum of Fmoc-p-PhAc-DAP (2c).................................. S15

Table S1. pH measurements of solutions of compounds 1, 1a, 1b, and 1c before and after triggering self-assembly by pH adjustment. S16

Table S2. Morphology and width of nanostructures observed in compounds 1, 1a, 1b, and 1c after 24 h of assembly.. S16

Figure S10. 1H NMR spectra of A) Fmoc-Phe (1) (10 mM) in unassembled monomeric form (DMSO-d_6), B) Fmoc-Phe (1) (10 mM) in D_2O with 15 mM NaOH, C) Fmoc-Phe (1) (10 mM) in D_2O 24 h after addition of 10 mM GdL (assembled), D) Fmoc-o-PhAc (1a) (10 mM) in unassembled monomeric form (DMSO-d_6), E) Fmoc-o-PhAc (1a) (10 mM) in D_2O with 15 mM NaOH, F) Fmoc-o-PhAc (1a) (10 mM) in D_2O 24 h after addition of 10 mM GdL (assembled), G) Fmoc-m-PhAc (1b) (10 mM) in unassembled monomeric form (DMSO-d_6), H) Fmoc-m-PhAc (1b) (10 mM) in D_2O with 15 mM NaOH, I) Fmoc-m-PhAc (1b) (10 mM) in D_2O 24 h after addition of 10 mM GdL (assembled), J) Fmoc-p-PhAc (1c) (10 mM) in unassembled monomeric form (DMSO-d_6), K) Fmoc-p-PhAc (1c) (10 mM) in D_2O with 15 mM NaOH, L) Fmoc-p-PhAc (1c) (10 mM) in D_2O 24 h after addition of 10 mM GdL (assembled).

Comparative integration to quantify monomer concentration was performed against an external standard of 24 mM DMF in DMSO-d_6 inserted in a sealed capillary tube. S17

Table S3. pH measurements of solutions of compounds 2, 2a, 2b, and 2c before and after triggering self-assembly by increasing solution ionic strength by NaCl addition...................... S18

Table S4. Morphology and width of compounds 2, 2a, 2b, and 2c in samples after 1h of assembly. S18

Figure S11. Digital images to estimate the critical gelation concentration of A) Fmoc-Phe-DAP (2) from 1 mM to 10 mM; B) Fmoc-o-PhAc-DAP (2a) at 10 mM, 15 mM and 20 mM; C) Fmoc-m-PhAc-DAP (2b) at 10 mM, 15 mM and 20 mM; and D) Fmoc-p-PhAc-DAP (2c) at 10 mM, 15 mM and 20 mM. For all cases, gelation was triggered by adding NaCl to a final concentration of 114 mM. S20

Figure S12. 1H NMR spectra of Fmoc-Phe-DAP (2) (10 mM) in A) DMSO-d_6 (unassembled), B) D_2O (partially assembled), C) D_2O with 10 mM NaCl (self-assembled), and D) D_2O with 114 mM NaCl. Comparative integration to quantify monomer concentration was performed against an external standard of 24 mM DMF in DMSO-d_6 inserted in a sealed capillary tube............. S21

Figure S13. 1H NMR spectra of Fmoc-o-PhAc-DAP (2b) (10 mM) in A) DMSO-d_6 (unassembled), B) D_2O (partially assembled), C) D_2O with 10 mM NaCl (self-assembled), and D) D_2O with 114 mM NaCl. Comparative integration to quantify monomer concentration was performed against an external standard of 24 mM DMF in DMSO-d_6 inserted in a sealed capillary tube. S22

Figure S14. 1H NMR spectra of Fmoc-m-PhAc-DAP (2b) (10 mM) in A) DMSO-d_6 (unassembled), B) D_2O (partially assembled), C) D_2O with 10 mM NaCl (self-assembled), and D) D_2O with 114 mM NaCl. Comparative integration to quantify monomer concentration was performed against an external standard of 24 mM DMF in DMSO-d_6 inserted in a sealed capillary tube. S23

Figure S15. 1H NMR spectra of Fmoc-p-PhAc-DAP (2c) (10 mM) in A) DMSO-d_6 (unassembled), B) D_2O (partially assembled), C) D_2O with 10 mM NaCl (self-assembled), and D) D_2O with 114 mM NaCl. Comparative integration to quantify monomer concentration was performed against an external standard of 24 mM DMF in DMSO-d_6 inserted in a sealed capillary tube. S24

Figure S16. TEM images and digital images of 10 mM aqueous solutions of A) Fmoc-Phe-DAP (2), B) Fmoc-o-PhAc-DAP (2a), C) Fmoc-m-PhAc-DAP (2b), and D) Fmoc-p-PhAc-DAP (2c). Compounds are dissolved in nanopure water with no added NaCl.. S25

Figure S17. TEM images and digital images of A) Fmoc-Phe-DAP (2), B) Fmoc-o-PhAc-DAP (2a), C) Fmoc-m-PhAc-DAP (2b), and D) Fmoc-p-PhAc-DAP (2c) at 10 mM in water with 10 mM NaCl. S26

Figure S18. TEM images and digital images of A) Fmoc-Phe-DAP (2), B) Fmoc-o-PhAc-DAP (2a), C) Fmoc-m-PhAc-DAP (2b), and D) Fmoc-p-PhAc-DAP (2c) at 10 mM in water with 25 mM NaCl. S27

Table S5. Experimental partition coefficient (log P) values of Fmoc-Phe and Fmoc-Phe-DAP isomers between octanol and water using the stir-flask method. Data was collected in triplicate with error reported as the standard deviation about the mean. S28

Figure S19. FTIR spectra of Fmoc-Phe-OH (1) neat powder, hydrogel (lyophilized, triggered with GdL), and pure GdL. (A) Full spectrum (3500–500 cm^{-1}). (B) Zoomed-in fingerprint region (2000–500 cm^{-1}) showing comparison with GdL. (C) Expanded view (1800–600 cm^{-1}) of neat powder and hydrogel, highlighting changes upon gelation. S29

Figure S20. FTIR spectra of Fmoc-o-PhAc (1a) neat powder, hydrogel (lyophilized, triggered with GdL), and pure GdL. (A) Full spectrum (3500–500 cm^{-1}). (B) Zoomed-in fingerprint region (2000–500 cm^{-1}) showing comparison with GdL. (C) Expanded view (1800–600 cm^{-1}) of neat powder and hydrogel, highlighting changes upon gelation. S30

Figure S21. FTIR spectra of Fmoc-m-PhAc (1b) neat powder, hydrogel (lyophilized, triggered with GdL), and pure GdL. (A) Full spectrum (3500–500 cm^{-1}). (B) Zoomed-in fingerprint region (2000–500 cm^{-1}) showing comparison with GdL. (C) Expanded view (1800–600 cm^{-1}) of neat powder and hydrogel, highlighting changes upon gelation. S31

Figure S22. FTIR spectra of Fmoc-p-PhAc (1c) neat powder, hydrogel (lyophilized, triggered with GdL), and pure GdL. (A) Full spectrum (3500–500 cm^{-1}). (B) Zoomed-in fingerprint region (2000–500 cm^{-1}) showing comparison with GdL. (C) Expanded view (1800–600 cm^{-1}) of neat powder and hydrogel, highlighting changes upon gelation. S32

Figure S23. FTIR spectra of Fmoc-Phe-DAP (2) neat powder, hydrogel (lyophilized, triggered with 114mM NaCl), (A) Full spectrum (3500–500 cm^{-1}). (B) Zoomed-in fingerprint region (2000–500 cm^{-1}) (C) Expanded view (1800–600 cm^{-1}) of neat powder and hydrogel, highlighting changes upon gelation. S33

Figure S24. FTIR spectra of Fmoc-o-PhAc-DAP (2) neat powder, hydrogel (lyophilized, triggered with 114mM NaCl), (A) Full spectrum (3500–500 cm^{-1}). (B) Zoomed-in fingerprint region (2000–500 cm^{-1}) (C) Expanded view (1800–600 cm^{-1}) of neat powder and hydrogel, highlighting changes upon gelation. S34

Figure S25. FTIR spectra of Fmoc-m-PhAc-DAP (2) neat powder, hydrogel (lyophilized, triggered with 114mM NaCl), (A) Full spectrum (3500–500 cm^{-1}). (B) Zoomed-in fingerprint region (2000–500 cm^{-1}) (C) Expanded view (1800–600 cm^{-1}) of neat powder and hydrogel, highlighting changes upon gelation. S35

Figure S26. FTIR spectra of Fmoc-p-PhAc-DAP (2) neat powder, hydrogel (lyophilized, triggered with 114mM NaCl), (A) Full spectrum (3500–500 cm^{-1}). (B) Zoomed-in fingerprint region (2000–500 cm^{-1}) (C) Expanded view (1800–600 cm^{-1}) of neat powder and hydrogel, highlighting changes upon gelation. S36

References S36

Synthesis of Fmoc-Phe-DAP isomers

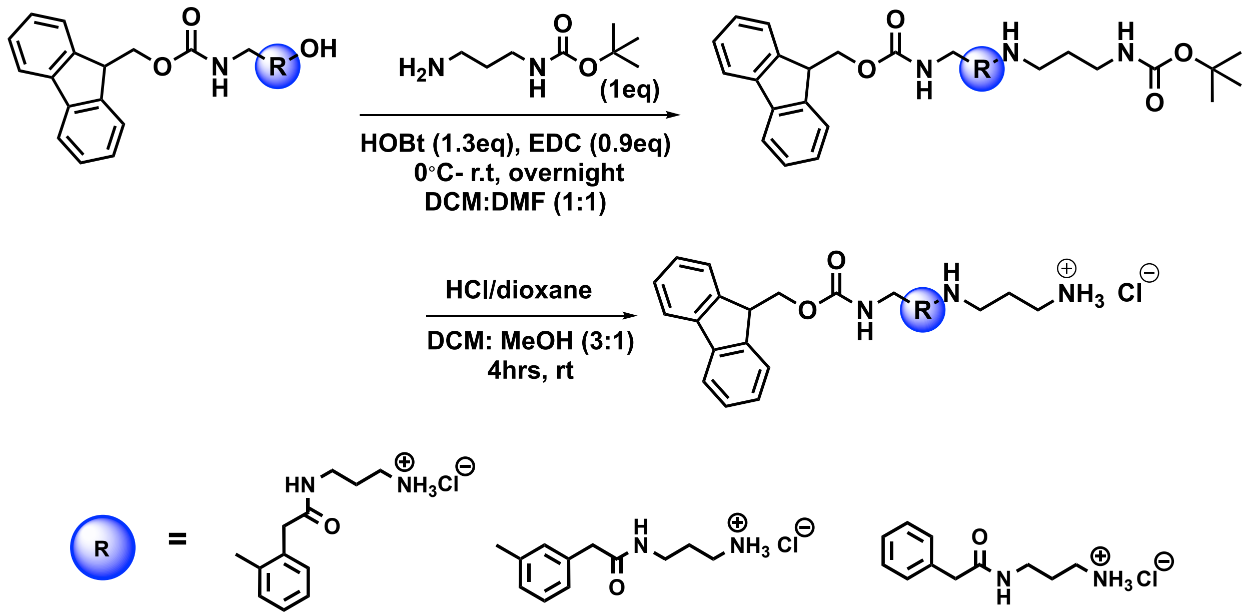

2a **2b** **2c**

Scheme S1. Synthesis of Fmoc-PhAc-DAP derivatives (**2a**, **2b**, and **2c**).

General procedure

The synthesis of Fmoc-Phe-DAP analogs were performed using the strategy outlined in **Scheme S1** above using an adaptation of our previously reported synthetic method.[1]

Fmoc-*o*-PhAc-DAP (**2a**):

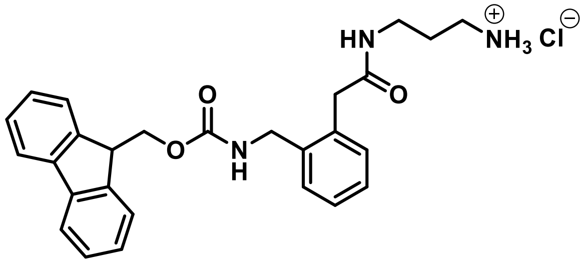

The product was prepared by following the same general procedure, with Fmoc-*o*-aminomethyl-phenylacetic acid (200 mg, 0.52 mmol) as starting material. The desired product was obtained as a white powder (131.3 mg, 0.27 mmol, 53% yield).

^{1}H NMR (500 MHz, DMSO-d$_6$) δ 8.28 (t, *J* = 5.8 Hz, 1H), 7.90 (d, *J* = 7.6 Hz, 2H), 7.82 (t, *J* = 5.9 Hz, 1H), 7.79 (s, 2H), 7.70 (d, *J* = 7.5 Hz, 2H), 7.42 (t, *J* = 7.5 Hz, 2H), 7.32 (t, *J* = 7.4 Hz, 2H), 7.26 – 7.15 (m, 4H), 4.33 (d, *J* = 7.0 Hz, 2H), 4.28 – 4.18 (m, 3H), 3.58 – 3.51 (m, 2H), 3.12 (q, *J* = 6.6 Hz, 2H), 2.76 (t, *J* = 7.5 Hz, 2H), 1.69 (p, *J* = 7.1 Hz, 2H) ppm; ^{13}C NMR (126 MHz, DMSO-d$_6$) δ 170.31, 156.04, 143.69, 140.57, 137.73, 134.01, 129.88, 127.43, 126.87, 126.66, 126.46, 125.01, 119.94, 65.21, 46.60, 41.40, 36.52, 35.61, 27.10 ppm. HRMS (ESI-TOF) (m/z) 444.2276 (444.2282 calcd for $C_{27}H_{30}N_3O_3$ [M]$^+$).

Fmoc-*m*-PhAc-DAP (**2b**):

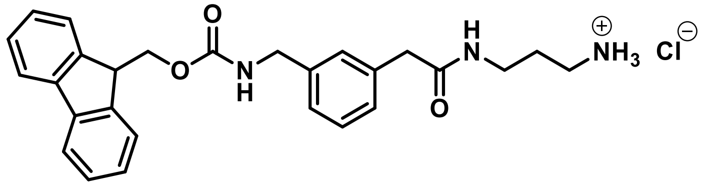

The product was prepared by following the same general procedure, with Fmoc-*m*-aminomethyl-phenylacetic acid (200 mg, 0.52 mmol) as N-terminal. The desired product was obtained as a white powder (143.3 mg, 0.29 mmol, 57% yield).

^{1}H NMR (500 MHz, DMSO-d$_6$) δ 8.24 (t, *J* = 5.8 Hz, 1H), 7.89 (t, *J* = 6.8 Hz, 3H), 7.77 (s, 3H), 7.70 (d, *J* = 7.5 Hz, 2H), 7.42 (t, *J* = 7.5 Hz, 2H), 7.33 (t, *J* = 7.4 Hz, 2H), 7.24 (d, *J* = 7.4 Hz, 1H), 7.14 (d, *J* = 7.7 Hz, 2H), 7.08 (d, *J* = 7.5 Hz, 1H), 4.33 (d, *J* = 7.0 Hz, 2H), 4.23 (t, *J* = 7.0 Hz, 1H), 4.16 (d, *J* = 6.2 Hz, 2H), 3.39 (s, 2H), 3.11 (q, *J* = 6.6 Hz, 2H), 2.75 (q, *J* = 6.6 Hz, 2H), 1.68 (q, *J* = 7.2 Hz, 2H) ppm; ^{13}C NMR (126 MHz, DMSO-d$_6$) δ 170.42, 156.34, 143.87, 140.73, 139.69, 136.29, 128.17, 127.72, 127.60, 127.42, 127.04, 125.17, 125.05, 120.10, 65.38, 46.75, 43.73, 42.28, 36.65, 35.70, 27.30. ppm. HRMS (ESI-TOF) (m/z) 444.2279 (444.2282 calcd for $C_{27}H_{30}N_3O_3$ [M]$^+$).

Fmoc-*p*-PhAc-DAP (**2c**):

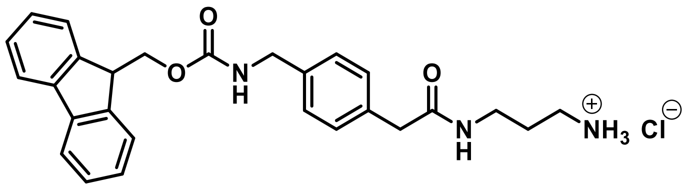

The product was prepared by following the same general procedure, with Fmoc-*p*-aminomethyl-phenylacetic acid (200 mg, 0.52 mmol) as N-terminal. The desired product was obtained as a white powder (170.6 mg, 0.36 mmol, 68% yield).

^{1}H NMR (500 MHz, DMSO-d$_6$) δ 8.23 (d, *J* = 5.7 Hz, 1H), 7.90 (d, *J* = 7.6 Hz, 2H), 7.84 (t, *J* = 6.2 Hz, 1H), 7.75 (s, 3H), 7.69 (d, *J* = 7.5 Hz, 2H), 7.42 (t, *J* = 7.4 Hz, 2H), 7.32 (t, *J* = 7.4 Hz, 2H), 7.19 (d, *J* = 7.9 Hz, 2H), 7.14 (d, *J* = 7.9 Hz, 2H), 4.34 (d, *J* = 7.0 Hz, 2H), 4.22 (t, *J* = 6.8 Hz, 1H), 4.14 (d, *J* = 6.2 Hz, 2H), 3.39 (s, 2H), 3.10 (q, *J* = 6.5 Hz, 2H), 2.75 (s, 2H), 1.67 (p, *J* = 7.0 Hz, 2H) ppm; ^{13}C NMR (126 MHz, DMSO-d$_6$) δ 170.55, 156.32, 143.88, 140.74, 137.81,

134.85, 128.84, 127.59, 126.91, 125.15, 120.11, 66.34, 65.30, 46.78, 43.48, 41.99, 36.65, 35.71, 27.30 ppm. HRMS (ESI-TOF) (m/z) 444.2280 (444.2282 calcd for $C_{27}H_{30}N_3O_3$ $[M]^+$).

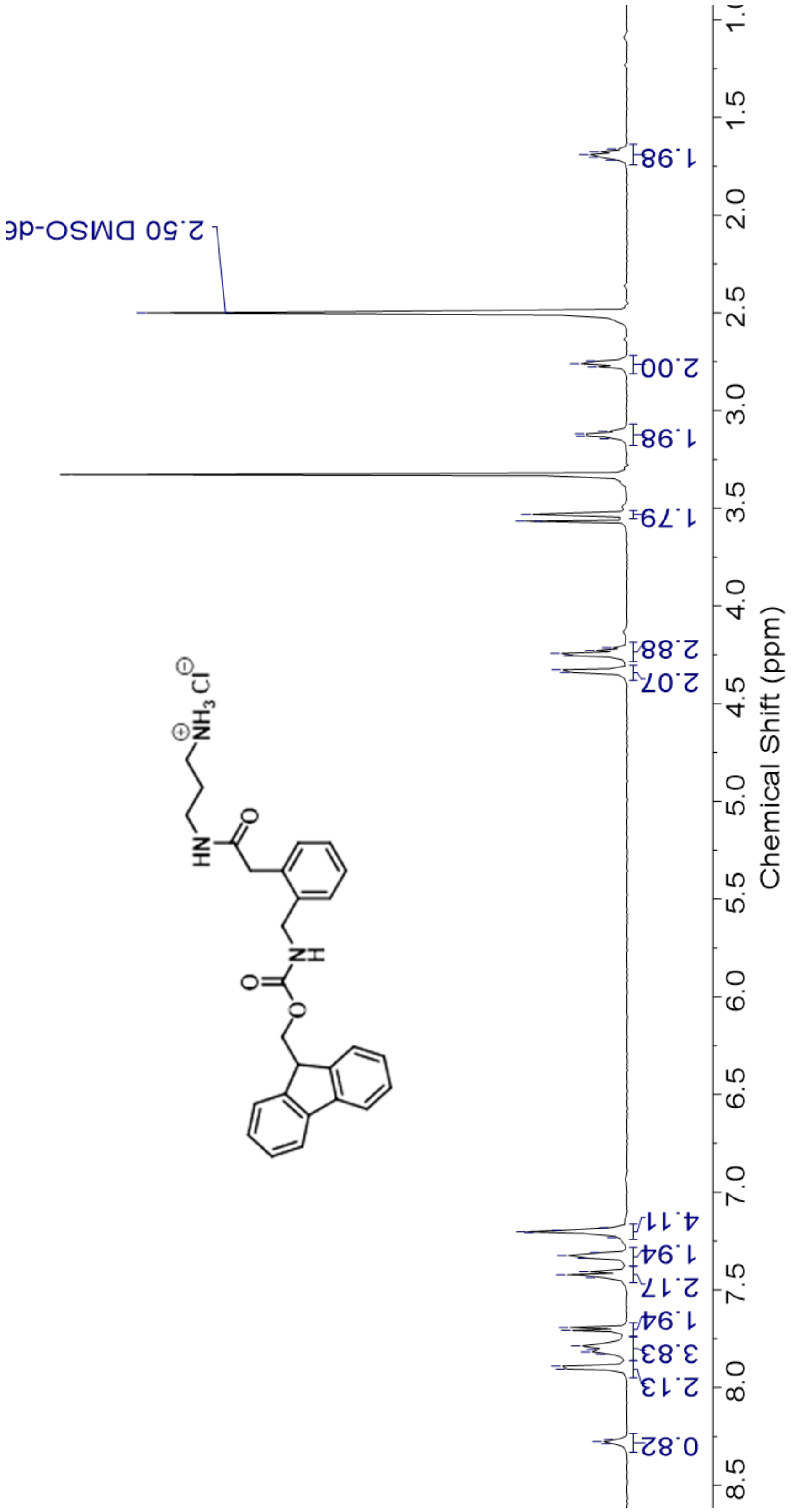

Figure S1. ^{1}H NMR spectrum of Fmoc-*o*-PhAc-DAP (**2a**).

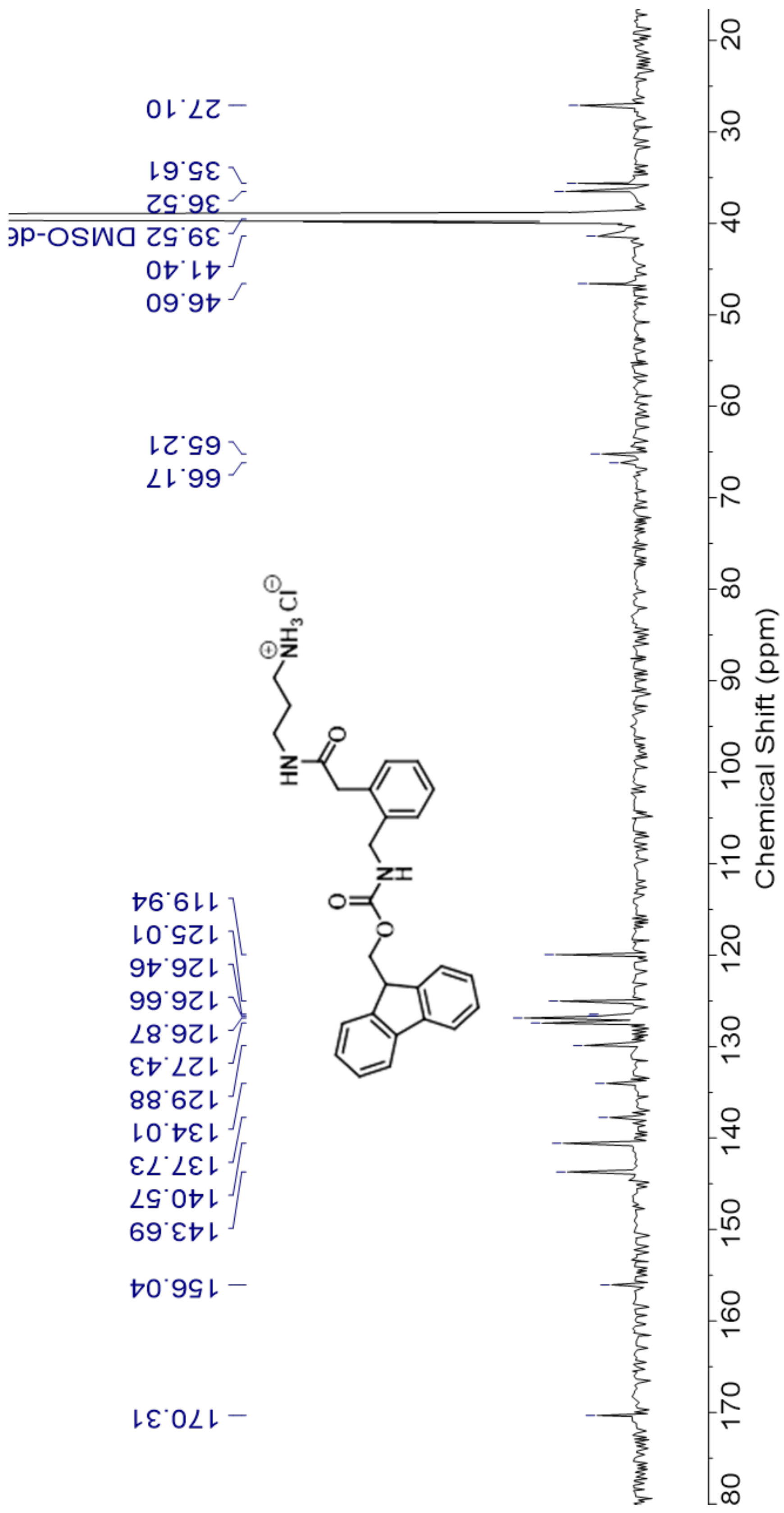

Figure S2. ^{13}C NMR spectrum of Fmoc-*o*-PhAc-DAP (**2a**).

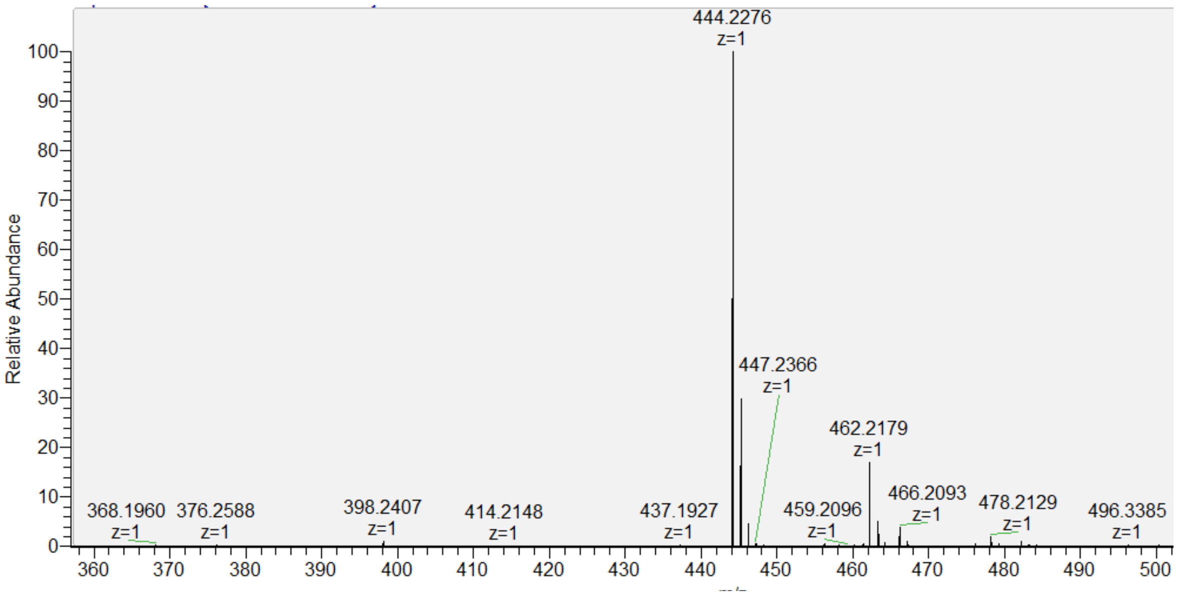

Figure S3. High-resolution mass spectrum of Fmoc-*o*-PhAc-DAP **(2a).**

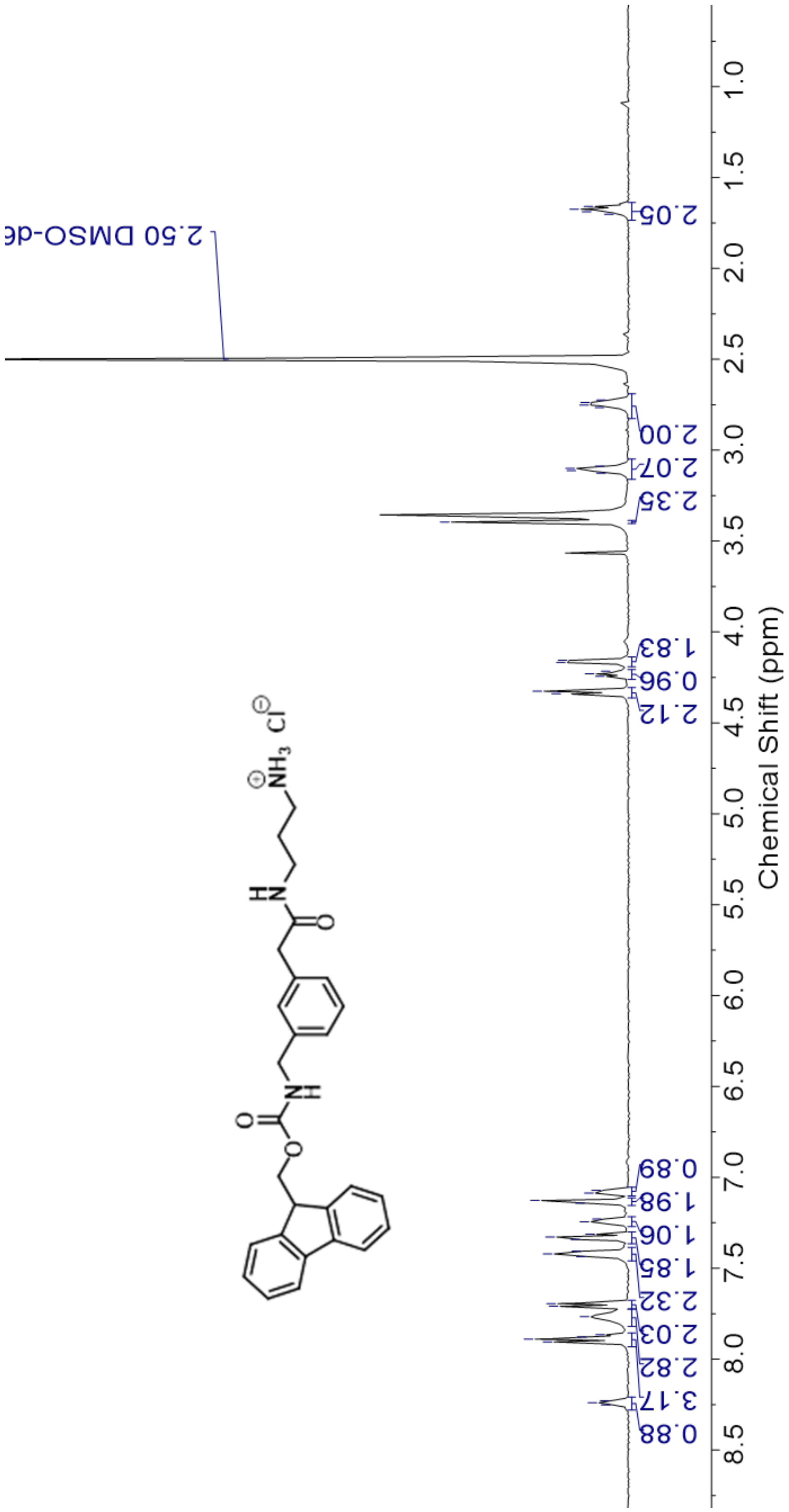

Figure S4. ^{1}H NMR spectrum of Fmoc-*m*-PhAc-DAP (**2b**).

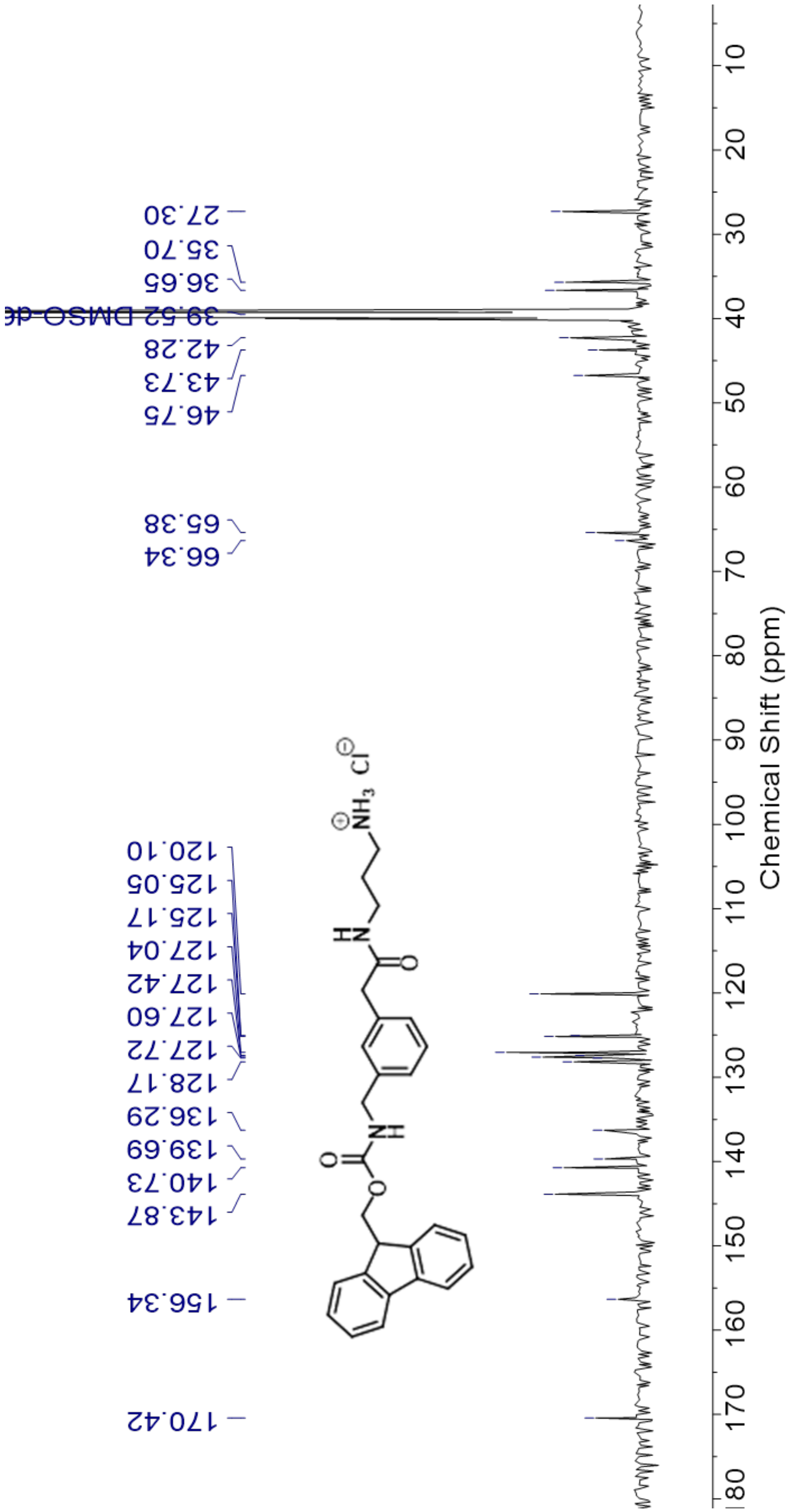

Figure S5. ^{13}C NMR spectrum of Fmoc-*m*-PhAc-DAP (**2b**).

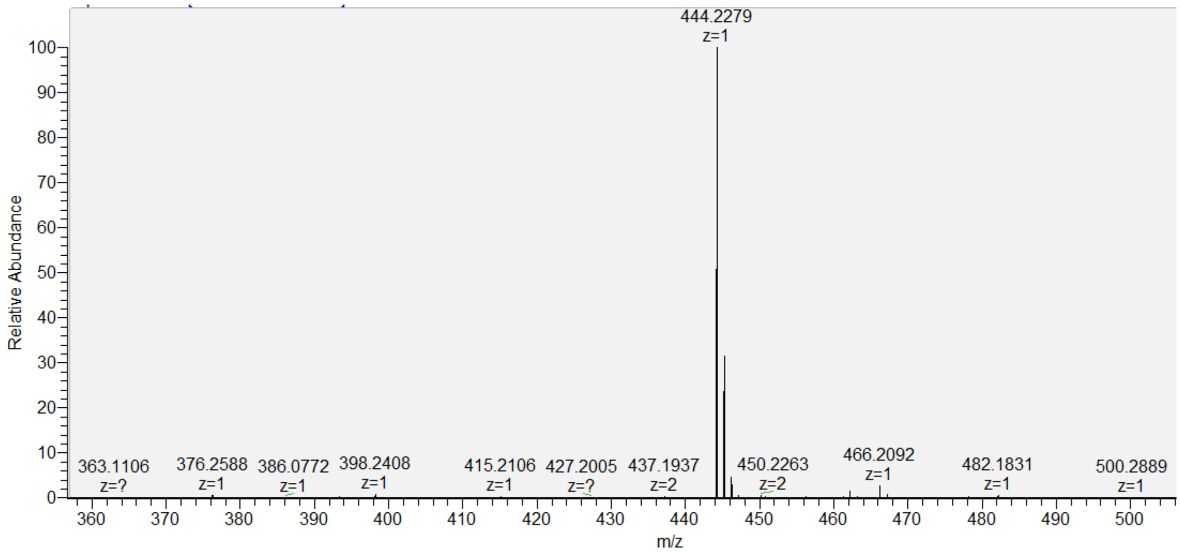

Figure S6. High-resolution mass spectrum of Fmoc-*m*-PhAc-DAP **(2b).**

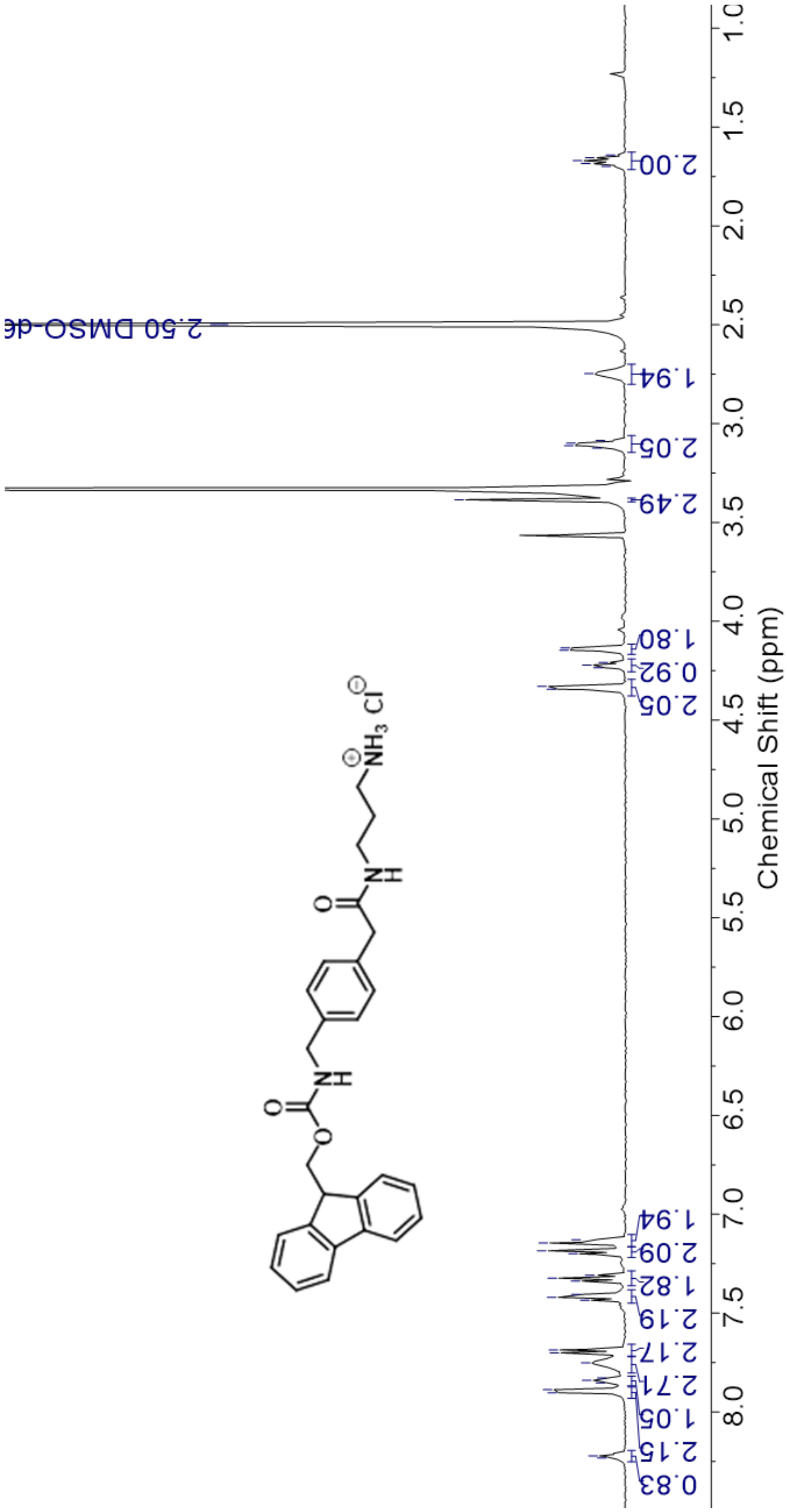

Figure S7. ^{1}H NMR spectrum of Fmoc-*p*-PhAc-DAP (**2c**).

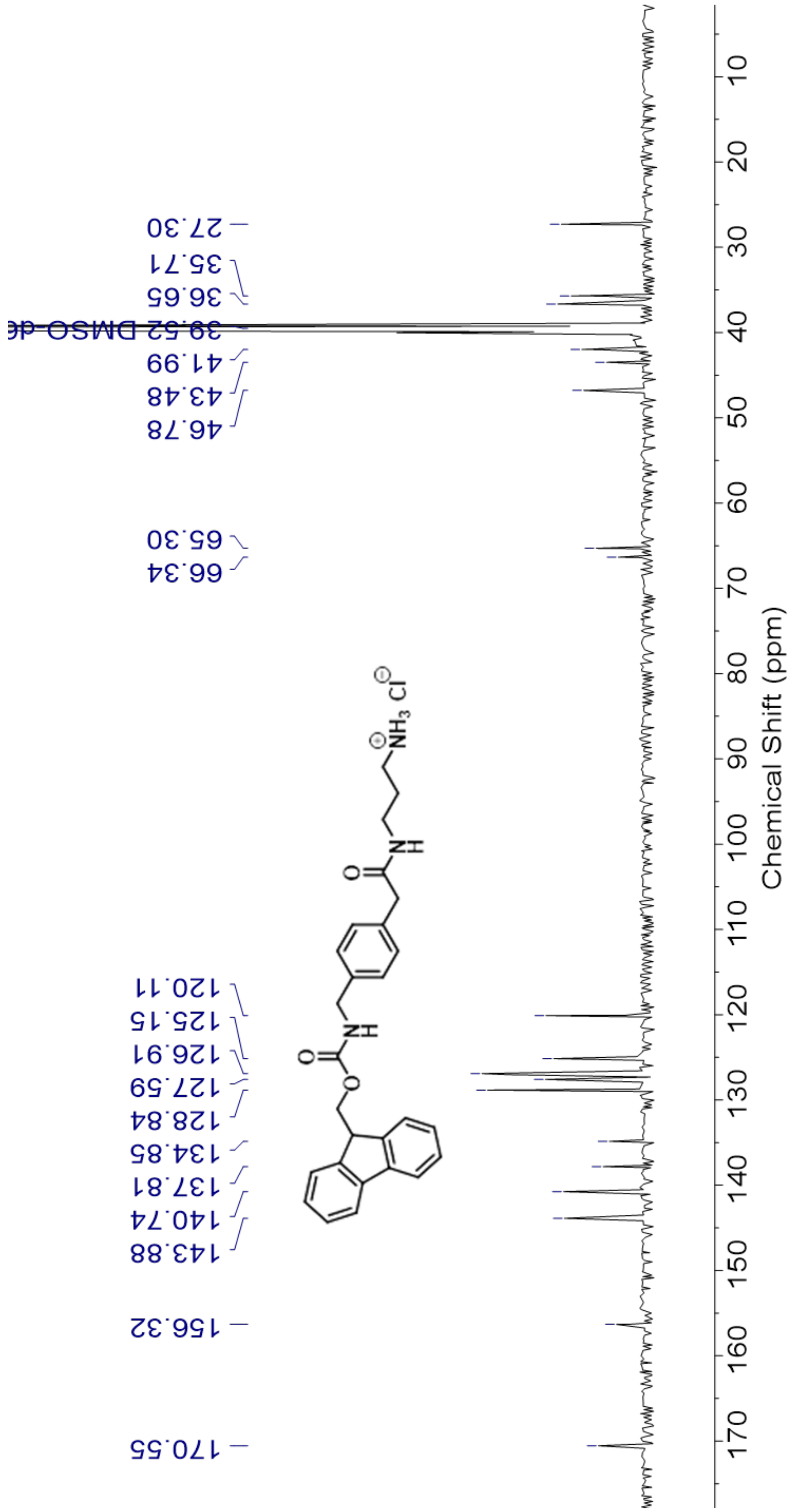

Figure S8. ^{13}C NMR spectrum of Fmoc-*p*-PhAc-DAP (**2c**).

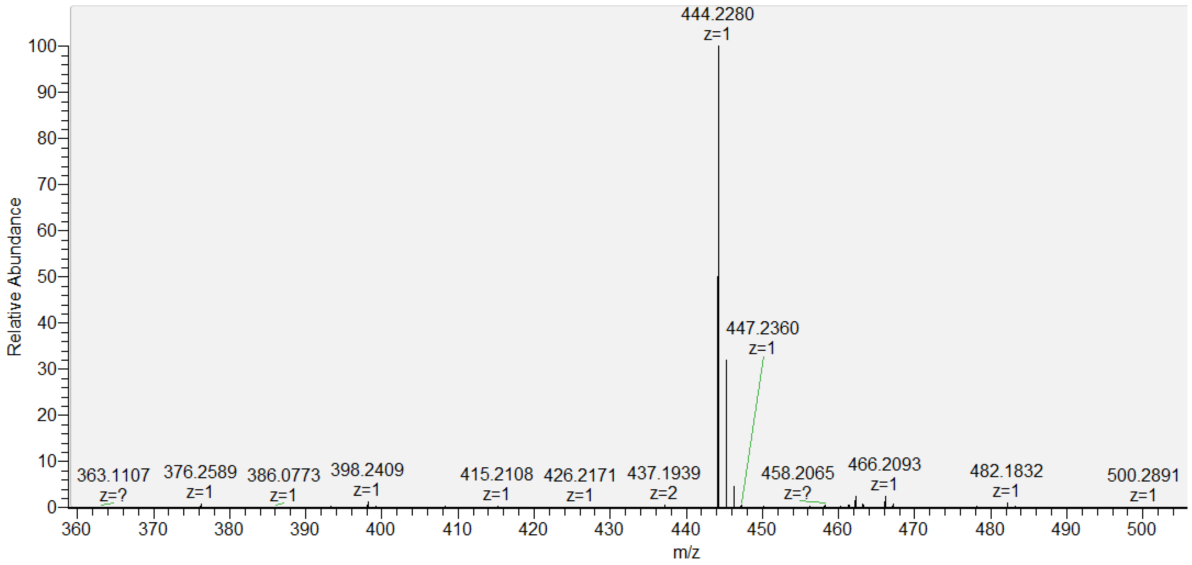

Figure S9. High-resolution mass spectrum of Fmoc-*p*-PhAc-DAP **(2c).**

Table S1. pH measurements of solutions of compounds **1, 1a, 1b**, and **1c** before and after triggering self-assembly by pH adjustment.

	**pH**			
**Conditions**	Fmoc-Phe (**1**)	Fmoc-*o*-PhAc (**1a**)	Fmoc-*m*-PhAc (**1b**)	Fmoc-*p*-PhAc (**1c**)
**In basic water (NaOH 15 mM)**	12.13 ± 0.04	11.91 ± 0.05	11.94 ± 0.03	11.84 ± 0.04
**After GdL addition (10 mM)**	6.45 ± 0.03	7.25 ± 0.05	8.19 ± 0.05	8.64 ± 0.01

Table S2. Morphology and width of nanostructures observed in compounds **1, 1a, 1b**, and **1c** after 24 h of assembly.

**Compound**	**Morphology**	**Width (nm)**
Fmoc-Phe (**1**)	nanoribbons/nanotubes	13.2 ± 2.6
Fmoc-*o*-PhAc (**1a**)	nanofibrils	10.5 ± 2.6
Fmoc-*m*-PhAc (**1b**)	clustered nano-pseudocrystals	190 ± 54.8
Fmoc-*p*-PhAc (**1c**)	nanosheet ribbons	59.8 ± 18.2

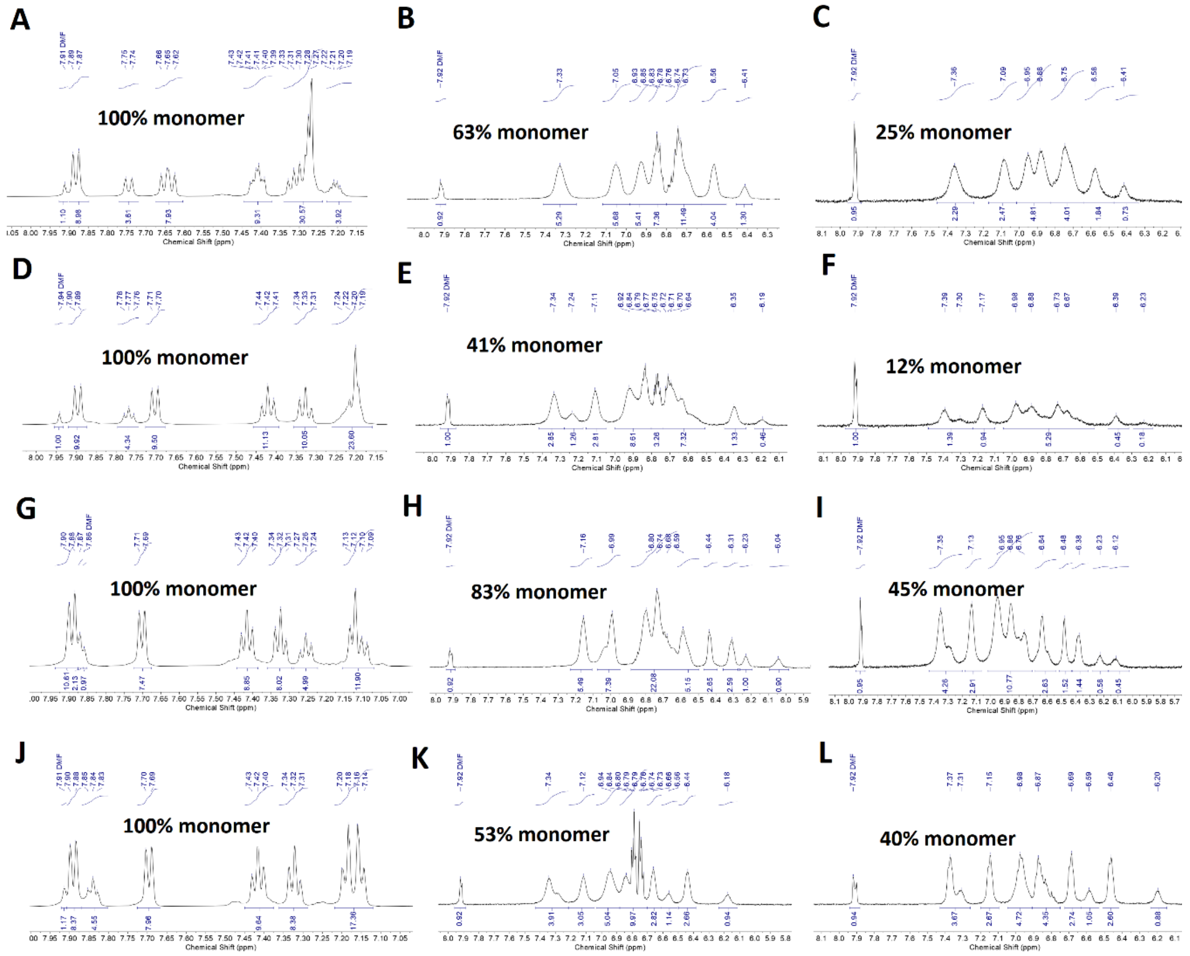

Figure S10. 1H NMR spectra of A) Fmoc-Phe (**1**) (10 mM) in unassembled monomeric form (DMSO-d_6), B) Fmoc-Phe (**1**) (10 mM) in D_2O with 15 mM NaOH, C) Fmoc-Phe (**1**) (10 mM) in D_2O 24 h after addition of 10 mM GdL (assembled), D) Fmoc-*o*-PhAc (**1a**) (10 mM) in unassembled monomeric form (DMSO-d_6), E) Fmoc-*o*-PhAc (**1a**) (10 mM) in D_2O with 15 mM NaOH, F) Fmoc-*o*-PhAc (**1a**) (10 mM) in D_2O 24 h after addition of 10 mM GdL (assembled), G) Fmoc-*m*-PhAc (**1b**) (10 mM) in unassembled monomeric form (DMSO-d_6), H) Fmoc-*m*-PhAc (**1b**) (10 mM) in D_2O with 15 mM NaOH, I) Fmoc-*m*-PhAc (**1b**) (10 mM) in D_2O 24 h after addition of 10 mM GdL (assembled), J) Fmoc-*p*-PhAc (**1c**) (10 mM) in unassembled monomeric form (DMSO-d_6), K) Fmoc-*p*-PhAc (**1c**) (10 mM) in D_2O with 15 mM NaOH, L) Fmoc-*p*-PhAc (**1c**) (10 mM) in D_2O 24 h after addition of 10 mM GdL (assembled). Comparative integration to quantify monomer concentration was performed against an external standard of 24 mM DMF in DMSO-d_6 inserted in a sealed capillary tube.

Table S3. pH measurements of solutions of compounds **2, 2a, 2b**, and **2c** before and after triggering self-assembly by increasing solution ionic strength by NaCl addition.

	**pH**			
**Conditions**	Fmoc-Phe-DAP (**2**)	Fmoc-*o*-PhAc-DAP (**2a**)	Fmoc-*m*-PhAc-DAP (**2b**)	Fmoc-*p*-PhAc-DAP (**2c**)
**In water**	6.44 ± 0.02	5.78 ± 0.02	5.57 ± 0.02	5.38 ± 0.04
**After NaCl 114 mM**	6.98 ± 0.05	7.06 ± 0.04	6.90 ± 0.03	7.03 ± 0.02

Table S4. Morphology and width of compounds **2, 2a, 2b,** and **2c** in samples after 1h of assembly.

**Compound**	**Self-assembly conditions**	**Morphology**	**Width (nm) [Length] (μm)**
Fmoc-Phe-DAP (**2**)	Water	nanoribbons	13.9 ± 5.7
	Water and 10 mM NaCl	nanoribbons	6.2 ± 1.5
	Water and 25 mM NaCl	nanoribbons	7.6 ± 4.5
	Water and 114 mM NaCl	nanoribbons	19.8 ± 10.6
Fmoc-*o*-PhAc-DAP (**2a**)	Water	nanotubes	179.8 ± 26.4 [3.9 ± 0.8] length
	Water and 10 mM NaCl	nanotubes	128.8 ± 33.7 [2.5 ± 0.5] length
	Water and 25 mM NaCl	nanotubes	186.5 ± 37.2 [1.9 ± 0.5] length
	Water and 114 mM NaCl	nanotubes	223 ± 50.9

			[2.5 ± 0.7] length
Fmoc-*m*-PhAc-DAP (**2b**)	Water	nanofibers	10.0 ± 2.2
	Water and 10 mM NaCl	nanofibers	14.7 ± 3.6
	Water and 25 mM NaCl	nanofibers	15.0 ± 8.3
	Water and 114 mM NaCl	nanofibers	12.2 ± 2.4
Fmoc-*p*-PhAc-DAP (**2c**)	Water	nanosheets	218.5 ± 95.3
	Water and 10 mM NaCl	nanosheets	150.1 ± 40.5
	Water and 25 mM NaCl	nanosheets	177.8 ± 49.5
	Water and 114 mM NaCl	nanosheets	158.2 ± 45.2

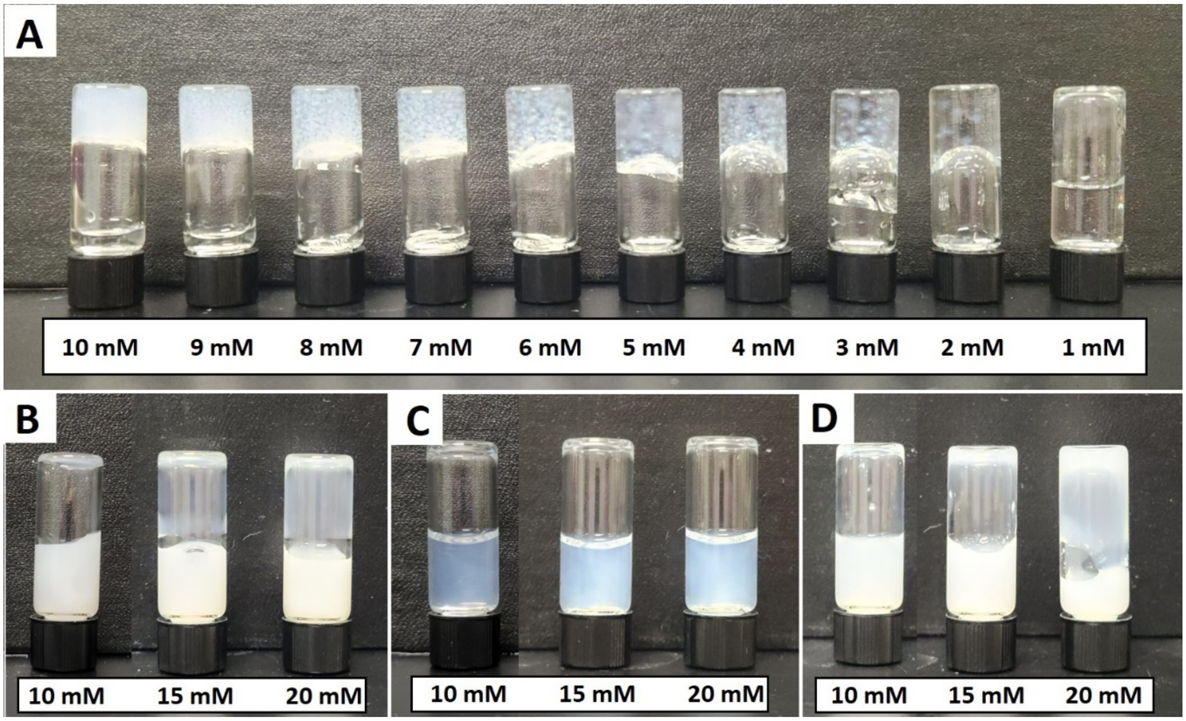

Figure S11. Digital images to estimate the critical gelation concentration of A) Fmoc-Phe-DAP (**2**) from 1 mM to 10 mM; B) Fmoc-*o*-PhAc-DAP (**2a**) at 10 mM, 15 mM and 20 mM; C) Fmoc-*m*-PhAc-DAP (**2b**) at 10 mM, 15 mM and 20 mM; and D) Fmoc-*p*-PhAc-DAP (**2c**) at 10 mM, 15 mM and 20 mM. For all cases, gelation was triggered by adding NaCl to a final concentration of 114 mM.

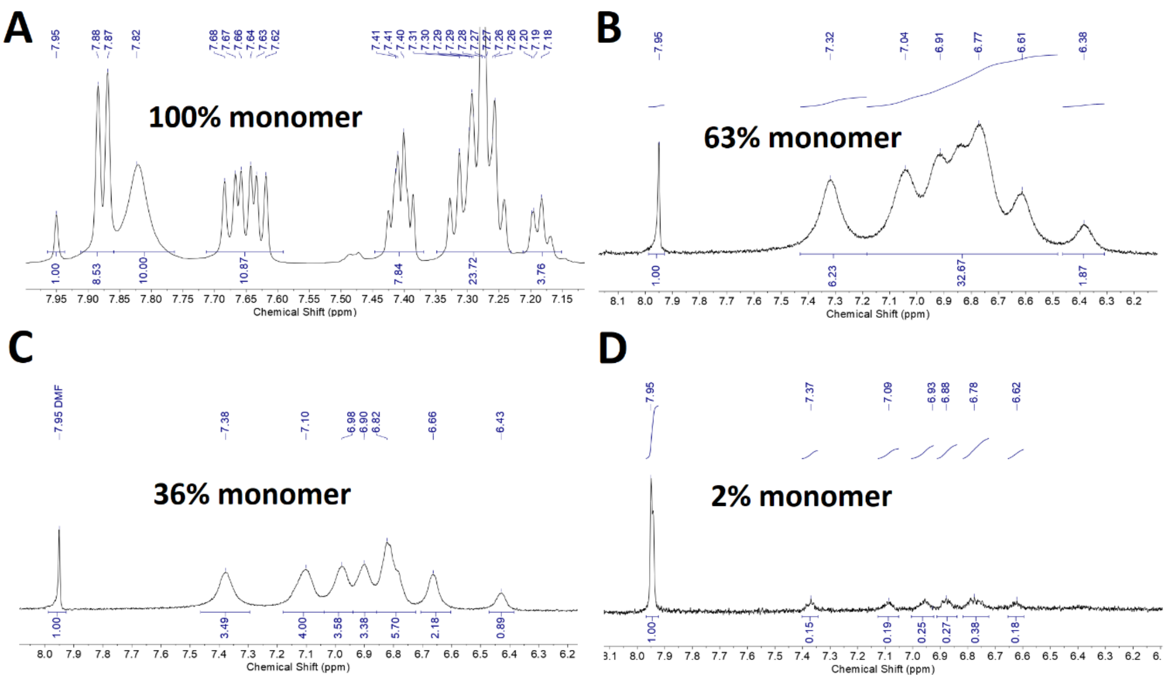

Figure S12. 1H NMR spectra of Fmoc-Phe-DAP (**2**) (10 mM) in A) DMSO-d_6 (unassembled), B) D_2O (partially assembled), C) D_2O with 10 mM NaCl (self-assembled), and D) D_2O with 114 mM NaCl. Comparative integration to quantify monomer concentration was performed against an external standard of 24 mM DMF in DMSO-d_6 inserted in a sealed capillary tube.

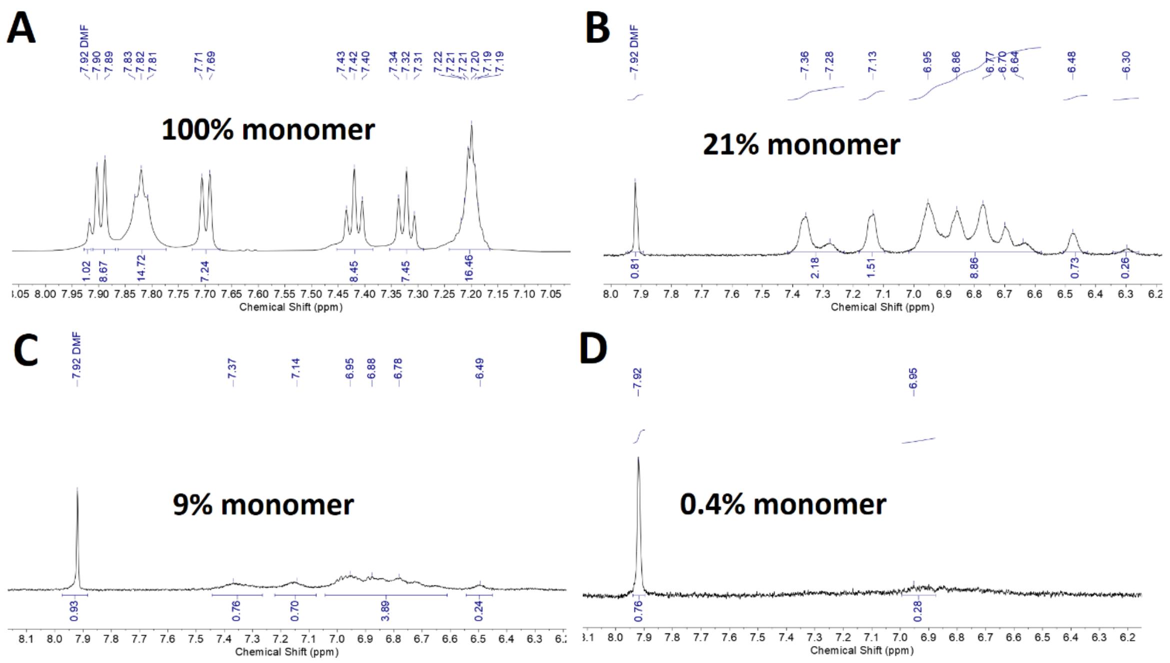

Figure S13. 1H NMR spectra of Fmoc-*o*-PhAc-DAP (**2b**) (10 mM) in A) DMSO-d_6 (unassembled), B) D_2O (partially assembled), C) D_2O with 10 mM NaCl (self-assembled), and D) D_2O with 114 mM NaCl. Comparative integration to quantify monomer concentration was performed against an external standard of 24 mM DMF in DMSO-d_6 inserted in a sealed capillary tube.

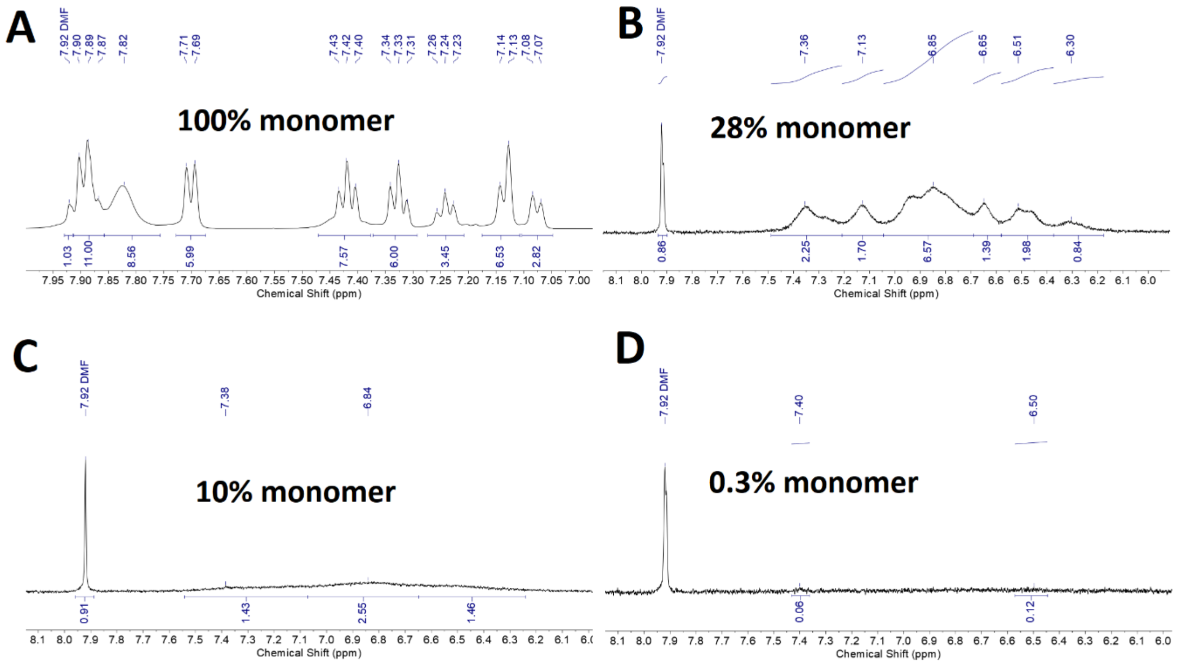

Figure S14. 1H NMR spectra of Fmoc-m-PhAc-DAP **(2b)** (10 mM) in A) DMSO-d_6 (unassembled), B) D_2O (partially assembled), C) D_2O with 10 mM NaCl (self-assembled), and D) D_2O with 114 mM NaCl. Comparative integration to quantify monomer concentration was performed against an external standard of 24 mM DMF in DMSO-d_6 inserted in a sealed capillary tube.

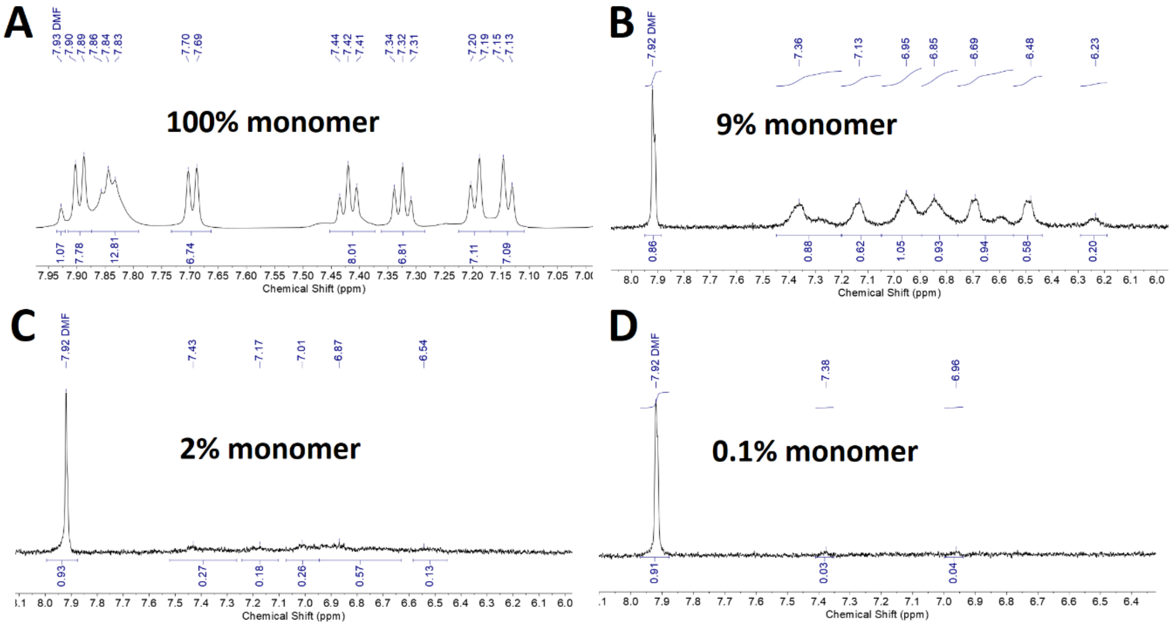

Figure S15. 1H NMR spectra of Fmoc-*p*-PhAc-DAP (**2c**) (10 mM) in A) DMSO-d_6 (unassembled), B) D_2O (partially assembled), C) D_2O with 10 mM NaCl (self-assembled), and D) D_2O with 114 mM NaCl. Comparative integration to quantify monomer concentration was performed against an external standard of 24 mM DMF in DMSO-d_6 inserted in a sealed capillary tube.

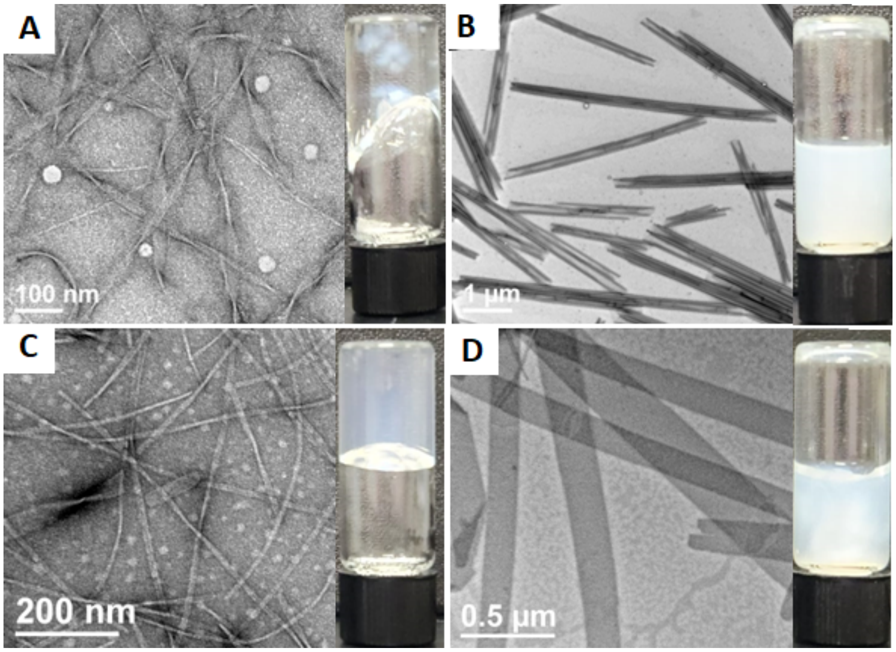

Figure S16. TEM images and digital images of 10 mM aqueous solutions of A) Fmoc-Phe-DAP (**2**), B) Fmoc-*o*-PhAc-DAP (**2a**), C) Fmoc-*m*-PhAc-DAP (**2b**), and D) Fmoc-*p*-PhAc-DAP (**2c**). Compounds are dissolved in nanopure water with no added NaCl.

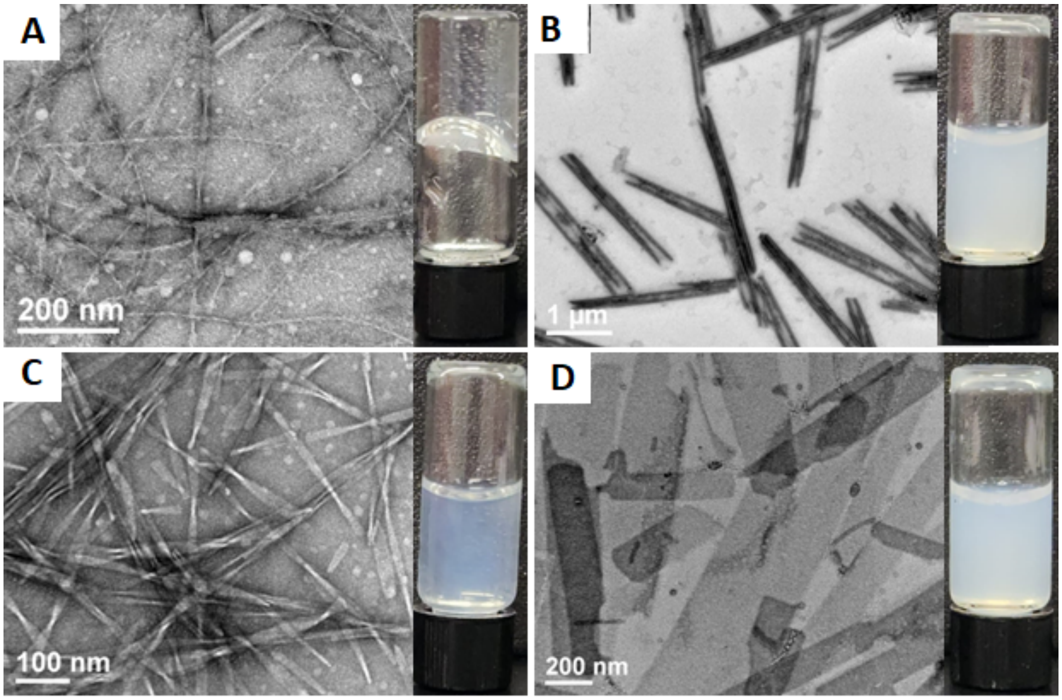

Figure S17. TEM images and digital images of A) Fmoc-Phe-DAP (**2**), B) Fmoc-*o*-PhAc-DAP (**2a**), C) Fmoc-*m*-PhAc-DAP (**2b**), and D) Fmoc-*p*-PhAc-DAP (**2c**) at 10 mM in water with 10 mM NaCl.

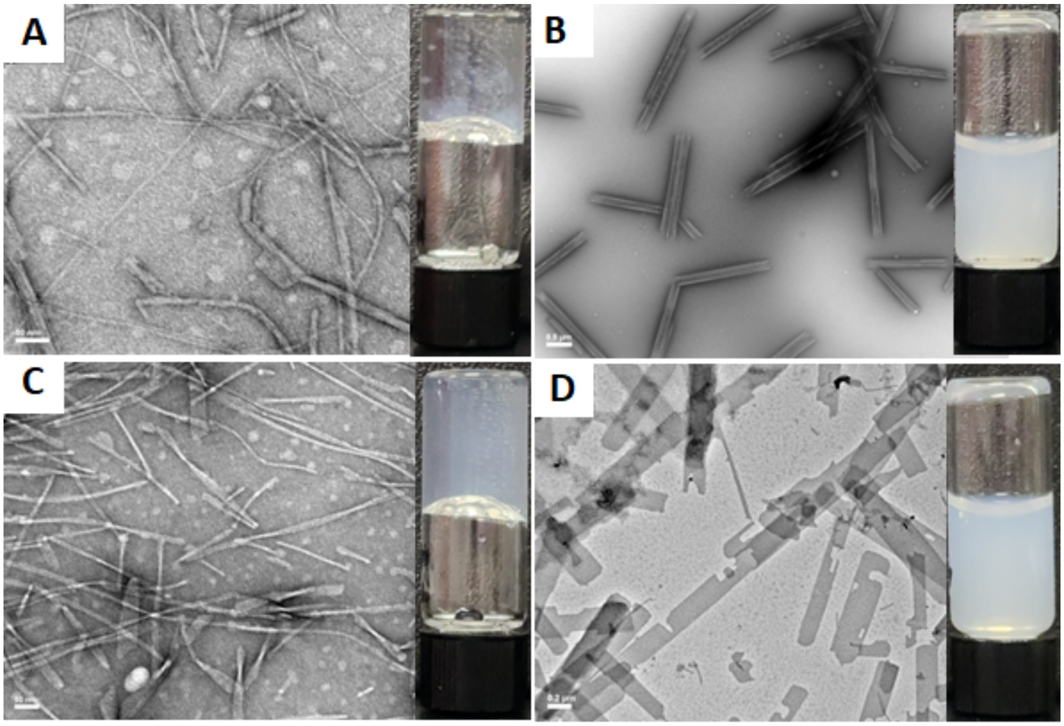

Figure S18. TEM images and digital images of A) Fmoc-Phe-DAP (2), B) Fmoc-*o*-PhAc-DAP (**2a**), C) Fmoc-*m*-PhAc-DAP (**2b**), and D) Fmoc-*p*-PhAc-DAP (**2c**) at 10 mM in water with 25 mM NaCl.

Table S5. Experimental partition coefficient (**log P)** values of Fmoc-Phe and Fmoc-Phe-DAP isomers between octanol and water using the stir-flask method. Data was collected in triplicate with error reported as the standard deviation about the mean.

**Compound**	**Experimental Log P**
Fmoc-Phe (**1**)	1.24 ± 0.02
Fmoc-*o*-PhAc (**1a**)	4.13 ± 0.03
Fmoc-*m*-PhAc (**1b**)	4.42 ± 0.02
Fmoc-*p*-PhAc (**1c**)	4.74 ± 0.1
Fmoc-Phe-DAP (**2**)	-0.10 ± 0.03
Fmoc-*o*-PhAc-DAP (**2a**)	-0.42 ± 0.01
Fmoc-*m*-PhAc-DAP (**2b**)	-0.56 ± 0.04
Fmoc-*p*-PhAc-DAP (**2c**)	-0.76 ± 0.04

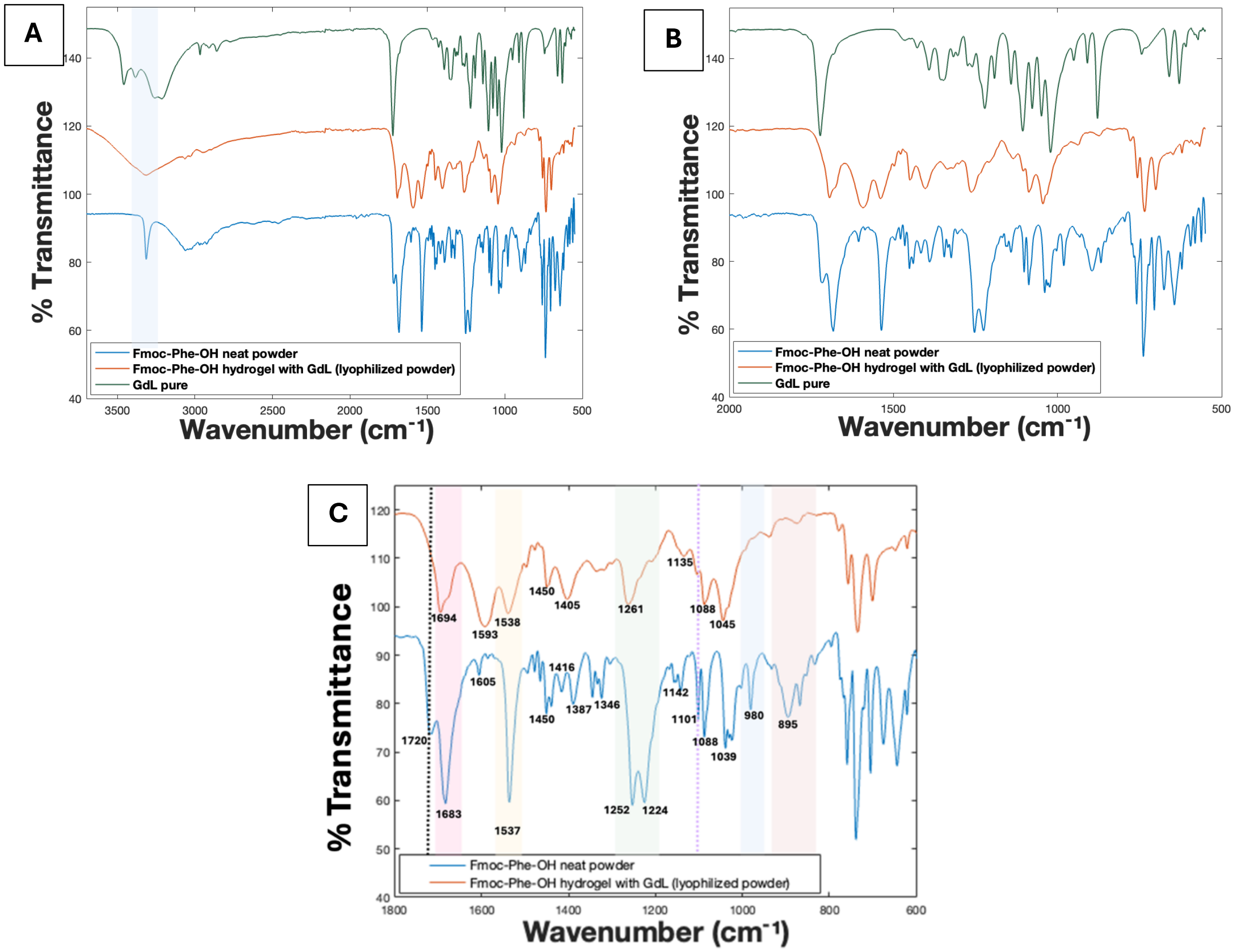

Figure S19. FTIR spectra of Fmoc-Phe-OH **(1)** neat powder, hydrogel (lyophilized, triggered with GdL), and pure GdL**. (A)** Full spectrum (3500–500 cm⁻¹). **(B)** Zoomed-in fingerprint region (2000–500 cm⁻¹) showing comparison with GdL. **(C)** Expanded view (1800–600 cm⁻¹) of neat powder and hydrogel, highlighting changes upon gelation.

.

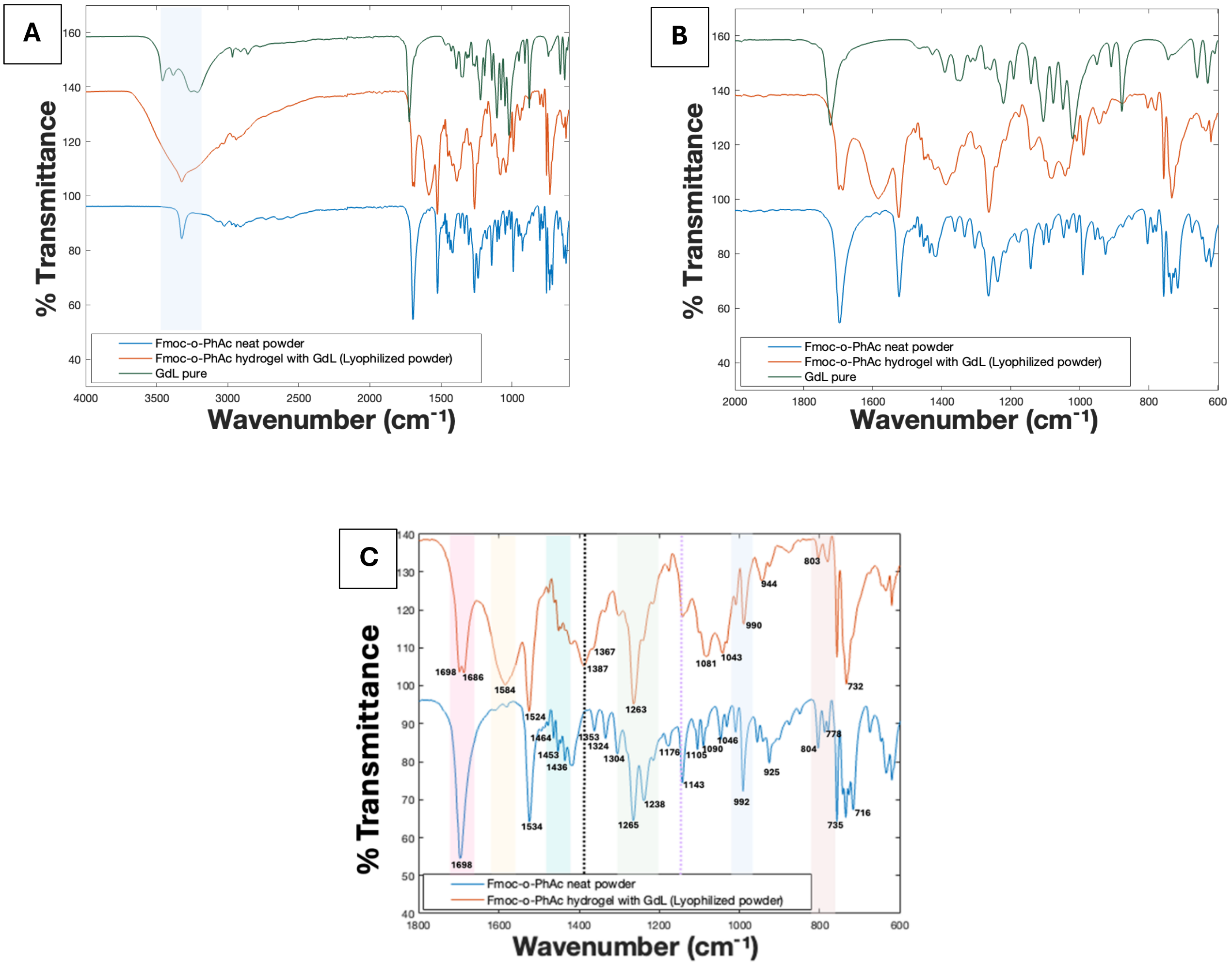

Figure S20. FTIR spectra of Fmoc-*o*-PhAc **(1a)** neat powder, hydrogel (lyophilized, triggered with GdL), and pure GdL**.** **(A)** Full spectrum (3500–500 cm^{-1}). **(B)** Zoomed-in fingerprint region (2000–500 cm^{-1}) showing comparison with GdL. **(C)** Expanded view (1800–600 cm^{-1}) of neat powder and hydrogel, highlighting changes upon gelation.

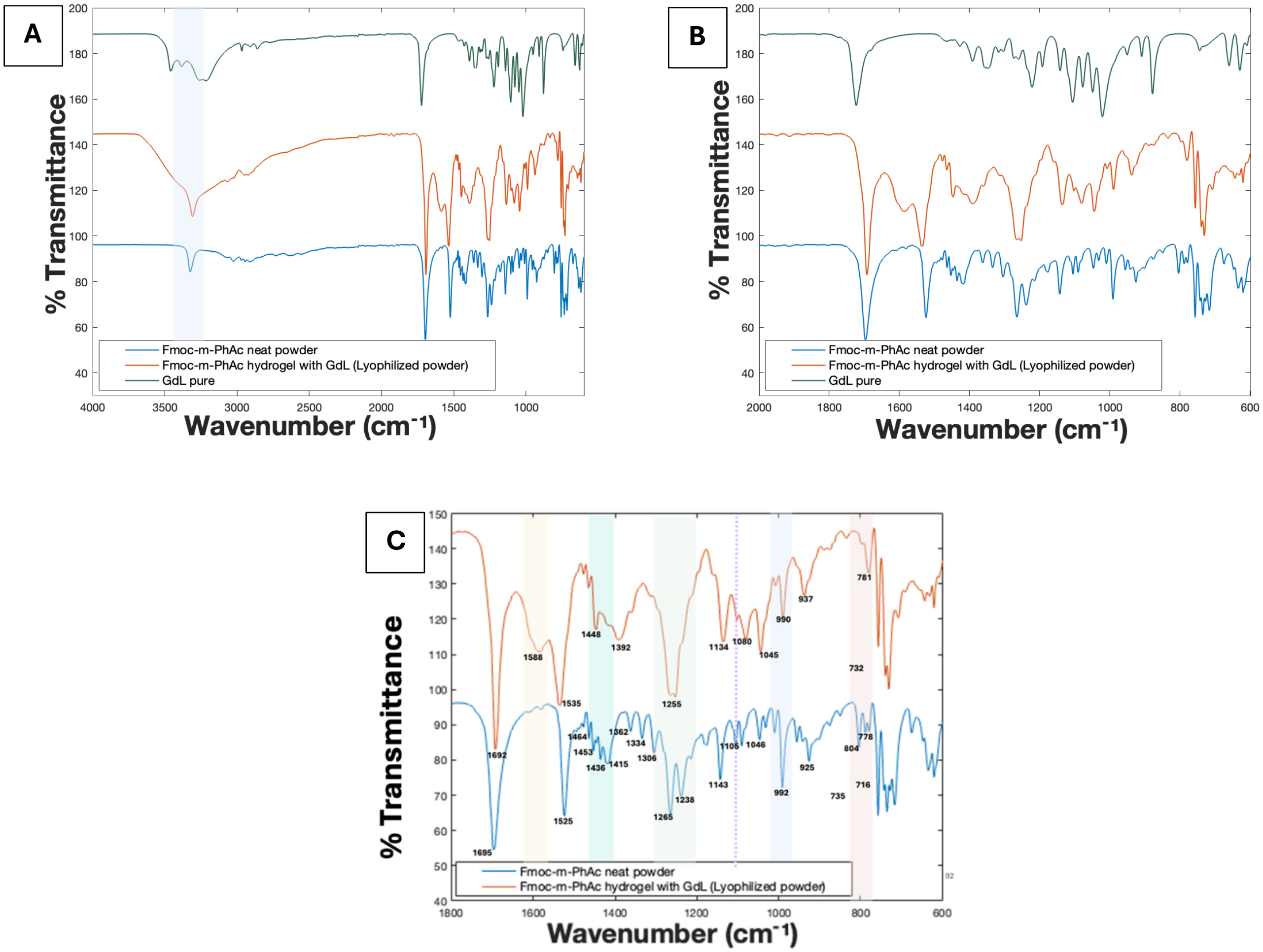

Figure S21. FTIR spectra of Fmoc-*m*-PhAc **(1b)** neat powder, hydrogel (lyophilized, triggered with GdL), and pure GdL**. (A)** Full spectrum (3500–500 cm^{-1}). **(B)** Zoomed-in fingerprint region (2000–500 cm^{-1}) showing comparison with GdL. **(C)** Expanded view (1800–600 cm^{-1}) of neat powder and hydrogel, highlighting changes upon gelation.

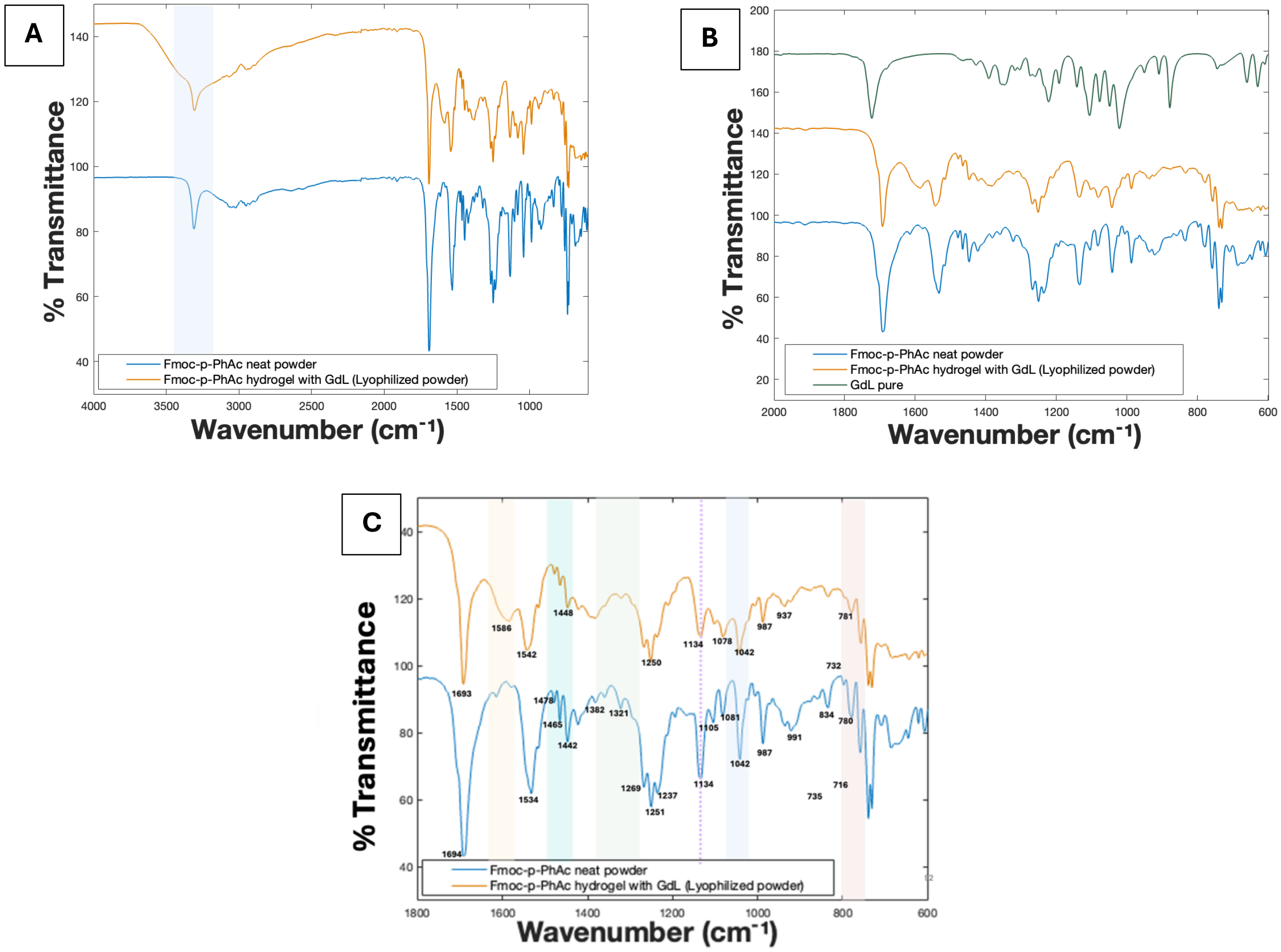

Figure S22. FTIR spectra of Fmoc-*p*-PhAc **(1c)** neat powder, hydrogel (lyophilized, triggered with GdL), and pure GdL**.** **(A)** Full spectrum (3500–500 cm^{-1}). **(B)** Zoomed-in fingerprint region (2000–500 cm^{-1}) showing comparison with GdL. **(C)** Expanded view (1800–600 cm^{-1}) of neat powder and hydrogel, highlighting changes upon gelation.

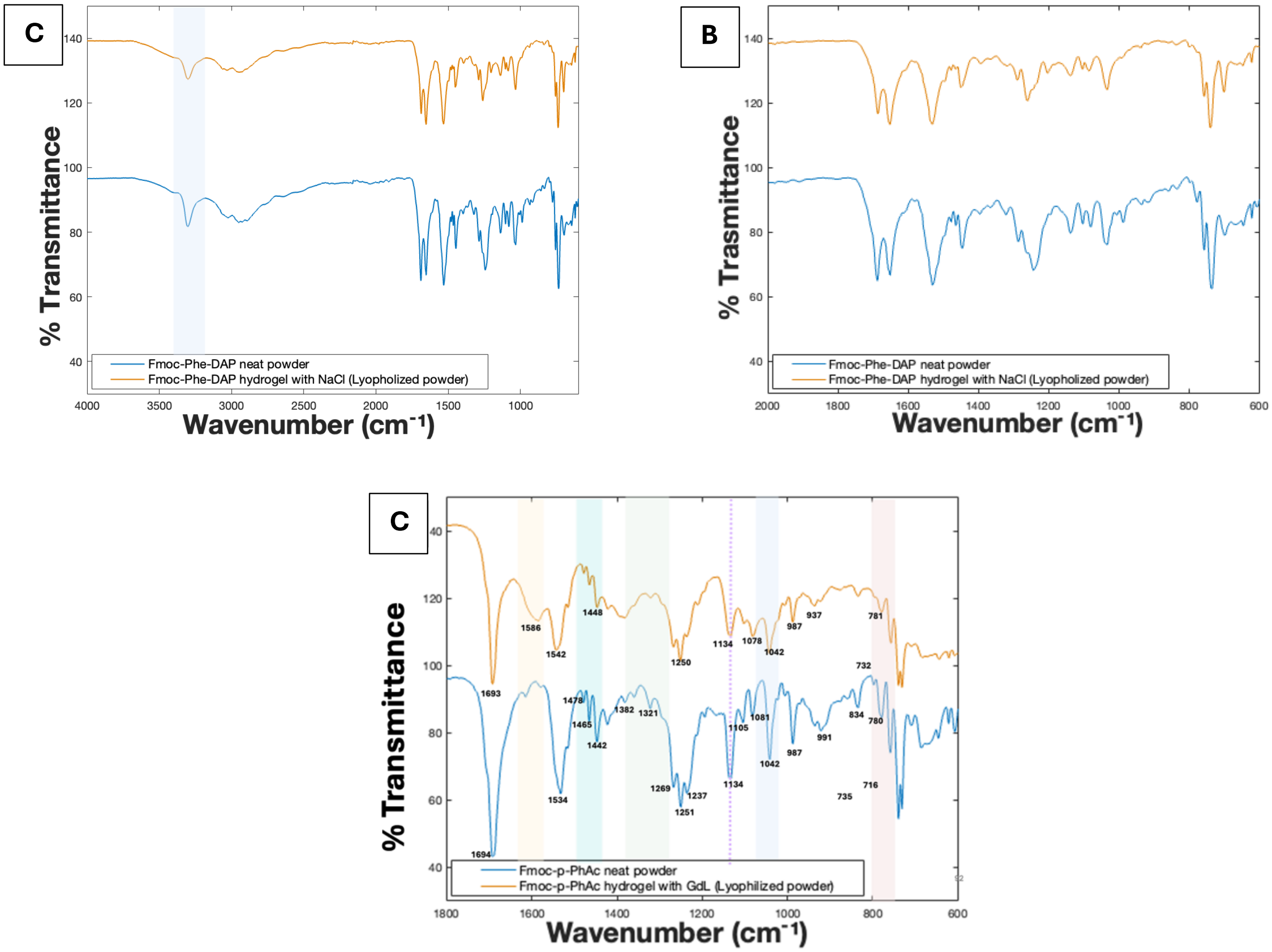

Figure S23. FTIR spectra of Fmoc-Phe-DAP **(2)** neat powder, hydrogel (lyophilized, triggered with 114mM NaCl), **(A)** Full spectrum (3500–500 cm⁻¹). **(B)** Zoomed-in fingerprint region (2000–500 cm⁻¹) **(C)** Expanded view (1800–600 cm⁻¹) of neat powder and hydrogel, highlighting changes upon gelation.

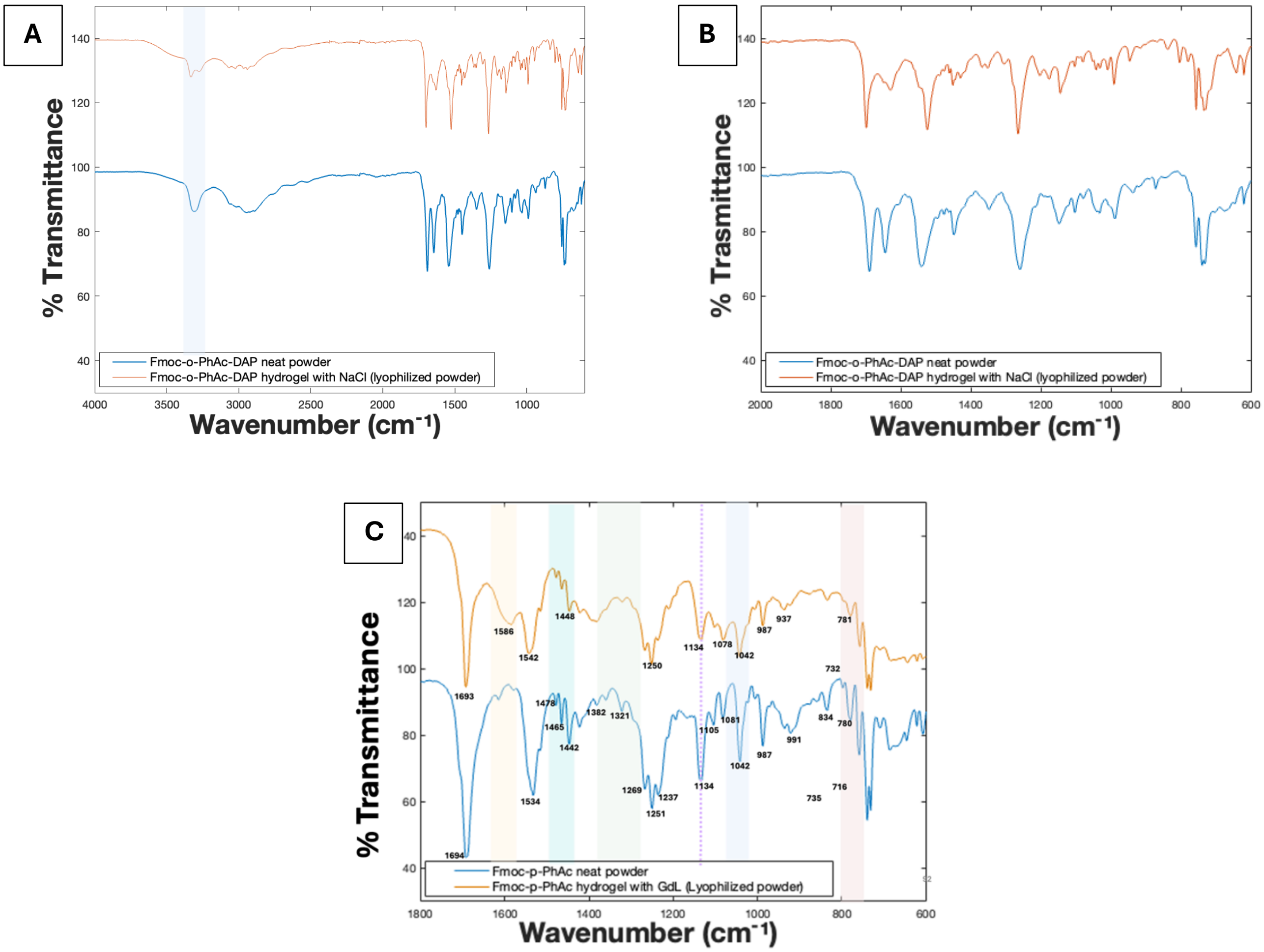

Figure S24. FTIR spectra of Fmoc-*o*-PhAc-DAP **(2)** neat powder, hydrogel (lyophilized, triggered with 114mM NaCl), **(A)** Full spectrum (3500–500 cm^{-1}). **(B)** Zoomed-in fingerprint region (2000–500 cm^{-1}) **(C)** Expanded view (1800–600 cm^{-1}) of neat powder and hydrogel, highlighting changes upon gelation.

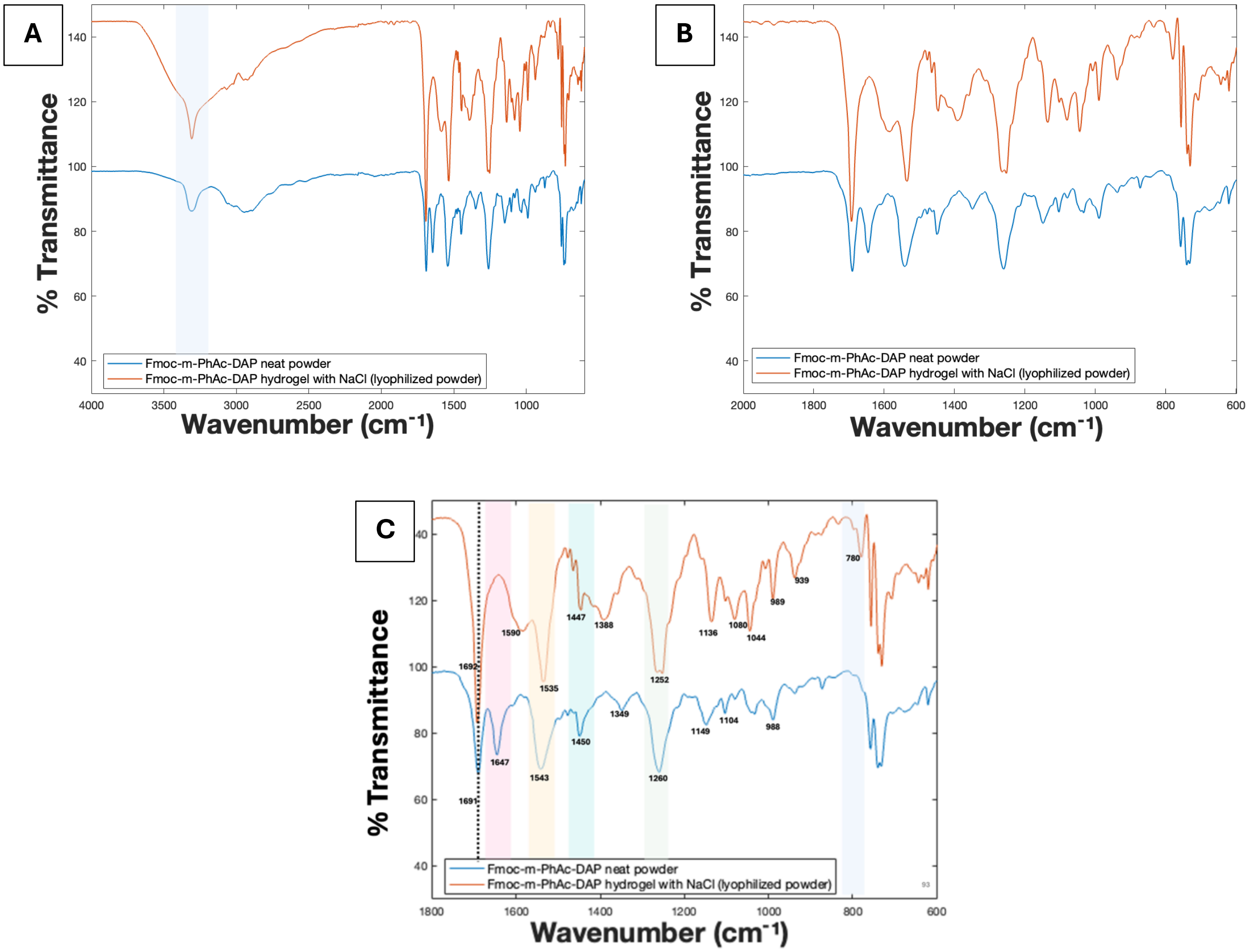

Figure S25. FTIR spectra of Fmoc-*m*-PhAc-DAP **(2)** neat powder, hydrogel (lyophilized, triggered with 114mM NaCl), **(A)** Full spectrum (3500–500 cm^{-1}). **(B)** Zoomed-in fingerprint region (2000–500 cm^{-1}) **(C)** Expanded view (1800–600 cm^{-1}) of neat powder and hydrogel, highlighting changes upon gelation.

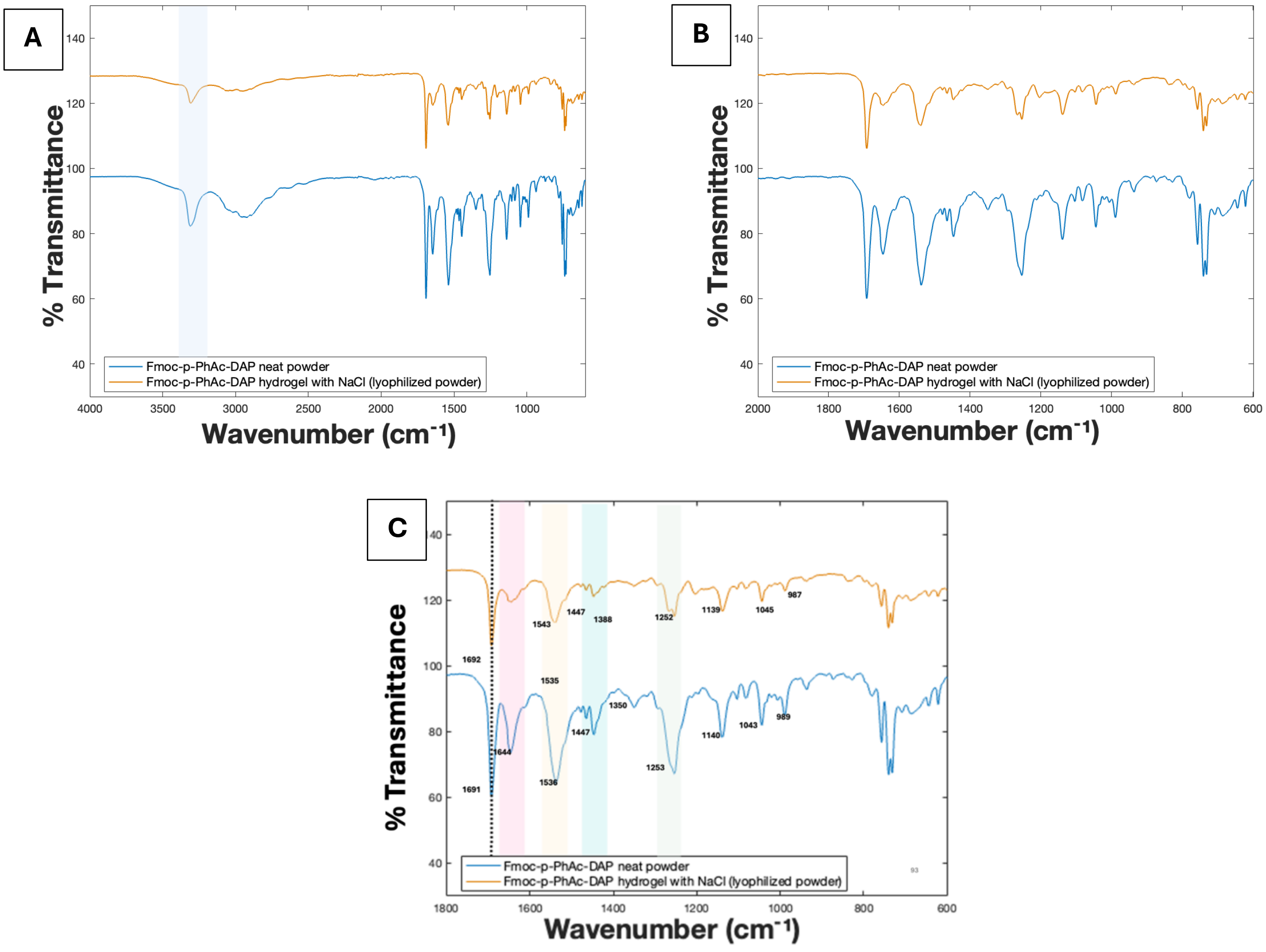

Figure S26. FTIR spectra of Fmoc-*p*-PhAc-DAP **(2)** neat powder, hydrogel (lyophilized, triggered with 114mM NaCl), **(A)** Full spectrum (3500–500 cm^{-1}). **(B)** Zoomed-in fingerprint region (2000–500 cm^{-1}) **(C)** Expanded view (1800–600 cm^{-1}) of neat powder and hydrogel, highlighting changes upon gelation.

References

(1) Abraham, B. L.; Toriki, E. S.; Tucker, N. D. J.; Nilsson, B. L. Electrostatic interactions regulate the release of small molecules from supramolecular hydrogels. *J. Mater. Chem. B* **2020**, *8* (30), 6366-6377. DOI: 10.1039/d0tb01157f.
